# Psychedelics promote plasticity by directly binding to BDNF receptor TrkB

**DOI:** 10.1038/s41593-023-01316-5

**Published:** 2023-06-05

**Authors:** Rafael Moliner, Mykhailo Girych, Cecilia A. Brunello, Vera Kovaleva, Caroline Biojone, Giray Enkavi, Lina Antenucci, Erik F. Kot, Sergey A. Goncharuk, Katja Kaurinkoski, Mirjami Kuutti, Senem M. Fred, Lauri V. Elsilä, Sven Sakson, Cecilia Cannarozzo, Cassiano R. A. F. Diniz, Nina Seiffert, Anna Rubiolo, Hele Haapaniemi, Elsa Meshi, Elina Nagaeva, Tiina Öhman, Tomasz Róg, Esko Kankuri, Marçal Vilar, Markku Varjosalo, Esa R. Korpi, Perttu Permi, Konstantin S. Mineev, Mart Saarma, Ilpo Vattulainen, Plinio C. Casarotto, Eero Castrén

**Affiliations:** 1 Neuroscience Center, HiLIFE, University of Helsinki, Helsinki, Finland; 2 Department of Pharmacology, Faculty of Medicine, University of Helsinki, Helsinki, Finland; 3 Department of Physics, Faculty of Science, University of Helsinki, Helsinki, Finland; 4 Institute of Biotechnology, Helsinki Institute of Life Science, University of Helsinki, Helsinki, Finland; 5 Department of Biomedicine, Faculty of Health, Aarhus University, Aarhus, Denmark; 6 Translational Neuropsychiatry Unit, Department of Clinical Medicine, Faculty of Health, Aarhus University, Aarhus, Denmark; 7 Department of Chemistry, Nanoscience Center, University of Jyväskylä, Jyväskylä, Finland; 8 Shemyakin-Ovchinnikov Institute of Bioorganic Chemistry, RAS, Moscow, Russia; 9 Moscow Institute of Physics and Technology, Dolgoprudny, Russia; 10 Department of Pharmacology, Ribeirão Preto Medical School, University of São Paulo, São Paulo, Brazil; 11 Neuroscience, University of Trieste, Trieste, Italy; 12 Biomedical Sciences, Hellenic University of Thessaloniki, Thessaloniki, Greece; 13 Molecular Basis of Neurodegeneration Unit, Instituto de Biomedicina de Valencia, CSIC, Valencia, Spain; 14 Department of Biological and Environmental Science, Nanoscience Center, University of Jyväskylä, Jyväskylä, Finland; 15 Structural and Quantitative Biology Research Program, Institute of Biotechnology, Instruct-HiLIFE, University of Helsinki, Helsinki, Finland; 16 Institute for Organic Chemistry and Chemical Biology, Center for Biomolecular Magnetic Resonance (BMRZ), Johann Wolfgang Goethe University, Frankfurt am Main, Germany

**Keywords:** Neurotrophic factors, Depression, Synaptic plasticity

## Abstract

Psychedelics produce fast and persistent antidepressant effects and induce neuroplasticity resembling the effects of clinically approved antidepressants. We recently reported that pharmacologically diverse antidepressants, including fluoxetine and ketamine, act by binding to TrkB, the receptor for BDNF. Here we show that lysergic acid diethylamide (LSD) and psilocin directly bind to TrkB with affinities 1,000-fold higher than those for other antidepressants, and that psychedelics and antidepressants bind to distinct but partially overlapping sites within the transmembrane domain of TrkB dimers. The effects of psychedelics on neurotrophic signaling, plasticity and antidepressant-like behavior in mice depend on TrkB binding and promotion of endogenous BDNF signaling but are independent of serotonin 2A receptor (5-HT_2A_) activation, whereas LSD-induced head twitching is dependent on 5-HT_2A_ and independent of TrkB binding. Our data confirm TrkB as a common primary target for antidepressants and suggest that high-affinity TrkB positive allosteric modulators lacking 5-HT_2A_ activity may retain the antidepressant potential of psychedelics without hallucinogenic effects.

## Abstract (web summary)

Moliner et al. show that psychedelics directly bind to the BDNF receptor TrkB with high affinity and promote BDNF-mediated plasticity and antidepressant-like effects, whereas their hallucinogenic-like effects are independent of TrkB binding.

## Main

Incidence of depression has surged during the past decade especially among young individuals^1^. There is therefore an urgent need for new, more efficient treatments for depression. Preliminary clinical trials suggest that psychedelics lysergic acid diethylamide (LSD) and psilocybin, through its metabolite psilocin (PSI), hold promise as fast-acting antidepressants with long-lasting therapeutic effects that are at least as effective as those of currently used antidepressants^2,3^. Until now, the acute hallucinogenic effects of psychedelics through activation of the serotonin 2A receptor (5-HT_2A_) have restricted their widespread clinical use, as they require specialized medical supervision during prolonged sessions set in a controlled clinical environment^4^. In addition, concerns exist that psychedelics may trigger hallucinogen persisting perception disorder (HPPD)^5^ or irreversible episodes of psychosis in susceptible populations, which has already led to the exclusion of patients with a family history of bipolar disorder or schizophrenia from participating in psychedelic clinical trials for depression^4,6^. However, recent reports suggest that the hallucinogenic effects of psychedelic compounds can be separated from their antidepressant-like and plasticity-promoting effects^7–9^, indicating that it may be possible to find compounds or treatment combinations that retain the antidepressant effects of psychedelics, but are devoid of the hallucinogenic effects. It is, however, unclear how these separate effects are mediated.

Essentially all antidepressant drugs, including psychedelics, promote neuroplasticity, which is considered a critical component of their therapeutic effect. Brain-derived neurotrophic factor (BDNF) and its receptor TrkB (neurotrophic receptor tyrosine kinase, Ntrk2) are central mediators of plasticity and the therapeutic action of antidepressants^10^. Recent findings show that antidepressants, including conventional antidepressants such as fluoxetine and imipramine, as well as the rapid-acting ketamine, directly bind to TrkB and allosterically potentiate BDNF signaling^11^. BDNF and TrkB have also been implicated in the action of psychedelics as downstream effectors of 5-HT_2A_ activation^12^. In this Article, because psychedelics produce robust spinogenesis and dendritogenesis^9,12^ and these effects are known to require intact TrkB signaling^12,13^, we set out to explore whether direct binding to TrkB might mediate the neuroplastic effects behind their therapeutic potential.

## Results

### Psychedelics are high-affinity TrkB ligands

**Fig. 1:**
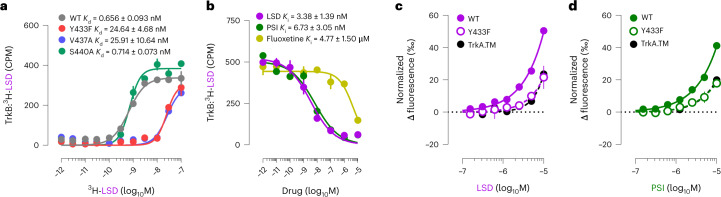
Psychedelics bind to TrkB. **a**, LSD binds with high affinity to WT TrkB, but binding is impaired by selective mutations in its binding pocket (Y433F and V437A). **b**, PSI and fluoxetine compete with LSD for TrkB binding. **c**,**d**, MST confirmed the interaction of LSD (**c**), and PSI (**d**), with TrkB, which is impaired by Y433F and TrkA.TM. CPM, counts per minute; M, molar. Data shown as mean ± s.e.m. Detailed statistics reported in Supplementary Table 1.

**Extended Data Fig. 1:**
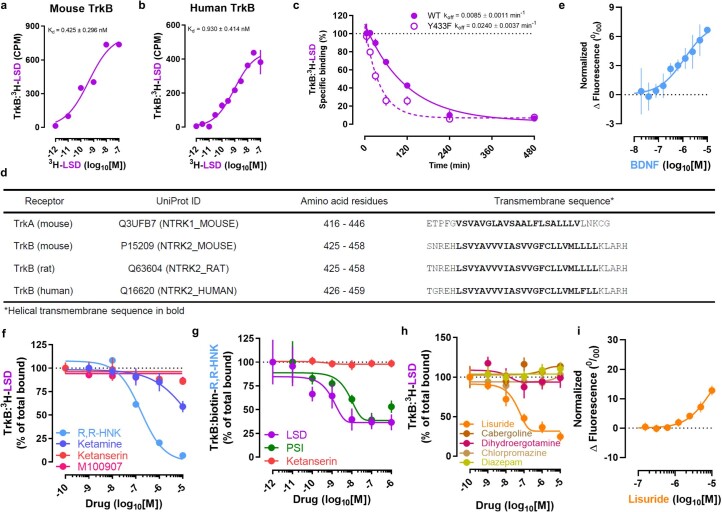
Psychedelics bind to TrkB. **a**, **b**, LSD binds with high affinity to mouse (**a**) and human (**b**) TrkB. **c**, LSD displays a slow dissociation constant from TrkB WT, but fast dissociation kinetics from Y433F. **d**, Protein sequences of TrkA and TrkB TMDs of different species. **e**, BDNF induces a shift in fluorescence of GFP-tagged TrkB in MST. **f**, Ketamine and R,R-HNK displace LSD from TrkB at high concentrations, but not ketanserin or M100907. **g**, Psychedelics, but not ketanserin, displace R,R-HNK binding to TrkB with nM concentrations. **h**, Lisuride displaces LSD from its TrkB binding site, but other LSD-related compounds (cabergoline, dihydroergotamine) fail to do so. Negative control compounds chlorpromazine and diazepam also fail to displace LSD. **i**, MST confirms lisuride binding to TrkB. Data shown as mean ± s.e.m, *P < 0.05. Detailed statistics reported in Supplementary Table 1.

We first tested whether psychedelics bind to TrkB in lysates of HEK293T cells transiently expressing TrkB and immunoprecipitated with a TrkB antibody. We observed that radiolabeled LSD (^3^H-LSD) binds directly to human (*K*_d_ = 0.930 ± 0.414 nM, *B*_max_ = 4.028 ± 0.326 pmol mg^−1^ protein), rat (*K*_d_ = 0.656 ± 0.093 nM, *B*_max_ = 3.053 ± 0.099 pmol mg^−1^ protein) and mouse TrkB (*K*_d_ = 0.425 ± 0.296 nM, *B*_max_ = 7.388 ± 0.771 pmol mg^−1^ protein) (Fig. 1a and Extended Data Fig. 1a,b,d) with high affinity, which is similar to that for its canonical target 5-HT_2A_ (*K*_i_ = 3.5 nM) (ref. ^14^). Remarkably, the affinity of LSD to TrkB was up to 1,000-fold higher than that of other antidepressants such as fluoxetine and ketamine to TrkB^11^. To verify the binding and to localize it, we tested the binding of ^3^H-LSD to a set of TrkB mutants (Fig. 1a). Replacement of the transmembrane domain (TMD) of TrkB with the corresponding sequence of TrkA (TrkA.TM) completely abolishes LSD binding, which restricts the LSD binding site to the TMD of TrkB. A tyrosine-to-phenylalanine point mutation (Y433F) in the TMD of TrkB that disrupts binding and plasticity-related effects of antidepressants also impairs LSD binding. V437A has similar effects, but S440A, a mutation that interferes with fluoxetine binding to TrkB^11^, did not influence LSD binding (Fig. 1a). These findings indicate that the LSD binding site in the TrkB TMD partially overlaps with that of other antidepressants. LSD displays a high residence time at TrkB (*k*_off_ = 0.0085 ± 0.0011 min^−1^), consistent with high affinity, but residence time is markedly reduced by Y433F (*k*_off_ = 0.0240 ± 0.0037 min^−1^) (Extended Data Fig. 1c).

We also found that PSI displaces ^3^H-LSD bound to TrkB with high affinity (*K*_i_ = 6.73 ± 3.05 nM, Fig. 1b), while fluoxetine (*K*_i_ = 4.77 ± 1.50 µM, Fig. 1b) and ketamine (*K*_i_ = 20.82 ± 8.86 µM, Extended Data Fig. 1f) display substantially lower TrkB affinities^11^. 2R,6R-hydroxynorketamine (R,R-HNK), an active metabolite of ketamine that binds to TrkB but shows low affinity to NMDA receptors^11,15^, displaces LSD with high nanomolar concentrations (*K*_i_ = 166.00 ± 21.15 nM, Extended Data Fig. 1f). In turn, biotinylated R,R-HNK is displaced from TrkB by psychedelics at low nanomolar concentrations (Extended Data Fig. 1g). To further define the TrkB binding specificity, we tested displacement of ^3^H-LSD by other ergoline compounds. We found that lisuride, a nonpsychedelic ergoline 5-HT_2A_ agonist, binds to TrkB at higher nanomolar concentrations in a linear manner, but other LSD-related compounds cabergoline and dihydroergotamine fail to displace ^3^H-LSD, as do the negative controls chlorpromazine and diazepam (Extended Data Fig. 1h,i). 5-HT_2A_ antagonists ketanserin and M100907 also fail to displace LSD binding to TrkB (Extended Data Fig. 1f,g). Importantly, no other known targets for psychedelics or antidepressants were identified by mass spectrometry (MS) in the samples used in our binding assays, confirming that LSD bound selectively to TrkB (Supplementary Table 2).

We confirmed binding to TrkB using microscale thermophoresis (MST) assay for unlabeled psychedelics to eGFP-tagged TrkB in lysates of HEK293T cells. MST confirmed that LSD, PSI and lisuride directly bind to native TrkB but not to Y433F or TrkA.TM mutants (Fig. 1c,d and Extended Data Fig. 1e). BDNF binding served as a positive control. The apparent potencies of all the compounds (including BDNF) were substantially lower when assayed with MST than the affinities obtained with binding assays, which may be due to interference by other lysate components.

**Fig. 2:**
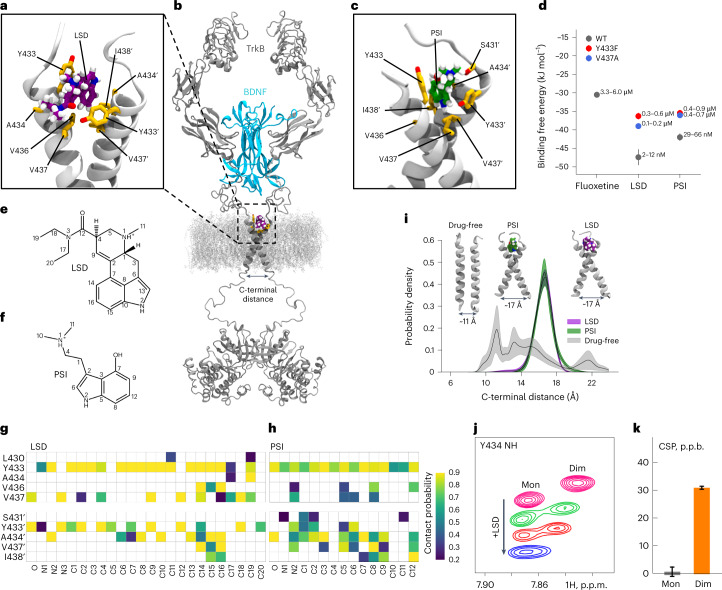
Characterization of the psychedelics binding site in the TrkB TMD. **a**–**c**, Representative MD snapshots showing the binding pocket for LSD (purple) (**a**) and PSI (green) (**c**) in the extracellular-facing crevice of the TrkB TMD dimer (gray). Side chains (yellow) of relevant binding site residues are displayed. A structural model of full-length TrkB dimer (gray) embedded in a lipid membrane is shown with bound BDNF (blue) and LSD (purple) (**b**). **d**, In silico binding free energy estimations for fluoxetine, LSD and PSI. Each free energy estimate (ΔG, circles) and its statistical error (SE, bars) were estimated from a separate set of FEP simulation (*n* = 1). Dissociation constants are given as a range with upper and lower bounds converted from ΔG − SE and ΔG + SE, respectively. **e**,**f**, Chemical structures of LSD (**e**) and PSI (**f**) with atom numbers annotated. **g**,**h**, Contact probability between binding pocket residues and LSD (**g**) or PSI (**h**). Residues of the second chain in the dimer are indicated with an apostrophe. **i**, Distributions of TMD dimer C-terminal distance show that LSD and PSI stabilize the cross-shaped conformation of TrkB favorable for receptor activation in a 40 mol% CHOL membrane. Lines represent the mean distribution, and bands represent the standard errors (*n* = 10 independent simulations). TMD conformations corresponding to indicated C-terminal distances and drug-bound states are shown in the inset. **j**, Expansions of overlaid ^1^H,^15^N-HSQC spectra of TrkB TMD in DMPC/DHPC bicelles showing the LSD-induced CSPs of resonance corresponding to Y433 amide group in the monomeric (Mon) and dimeric (Dim) states. p.p.m., parts per million. **k**, CSPs of the Y433 amide group in Mon and Dim states induced by the addition of LSD. p.p.b., parts per billion (10 p.p.b. = 0.01 p.p.m.). Data shown as mean ± s.e.m. Detailed statistics reported in Supplementary Table 1.

**Extended Data Fig. 2:**
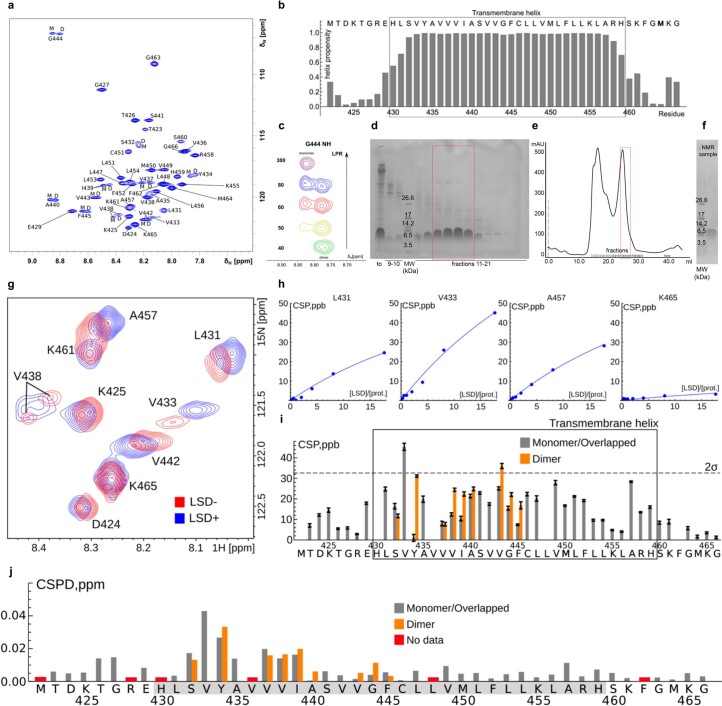
NMR confirms LSD binding to TrkB TMD. **a**, ^1^H,^15^N-TROSY-HSQC spectrum of TrkB TMD in DMPC/DHPC bicelles. Spectrum was recorded at lipid-to-protein ratio (LPR) 80. Assigned are the signals of backbone amide groups. For splitted signals M and D stand for monomeric and dimeric forms of TrkB TMD, respectively. **b**, Propensity of alpha-helical structure of TrkB TMD calculated by TALOS-N software based on the NMR chemical shifts. Rectangle shows the transmembrane helix. **c**, Appearance of the cross-peak corresponding to the G444 amide group in ^1^H,^15^N-HSQC spectrum of TrkB TMD at various LPR**s**. Two states are clearly visible, the population of states depends on the LPR. LPR 80 corresponds to the equal intensity of cross-peaks, corresponding to the dimer and monomer of the protein. **d**, Purification of TrkB TMD by size-exclusion chromatography. SDS-PAGE analysis of TrkB TMD purification. to, protein sample before purification; MW, molecular weight marker (kDa); 9–10 and 11–21, fractions. **e**, Size-exclusion chromatography profile. Fractions of purified protein used for following sample preparation shown boxed in red. **f**, SDS-PAGE analysis of final NMR sample of TrkB TMD. **g**, Overlay of ^1^H,^15^N-HSQC spectra of TrkB TMD in phospholipid DMPC/DHPC bicelles recorded in absence (LSD-) and presence of ligand (LSD+). **h**, CSPs of several amide groups of TrkB are plotted as a function of the LSD-to-protein ratio. **i**, CSPs of amide groups found for TrkB TMD upon LSD binding. The CSPs of V433, Y434 and V443 are the most pronounced upon LSD addition, indicating that the LSD binding interface is close to the N-terminus of the transmembrane helix. Transmembrane helix is indicated according to the NMR chemical shift analysis in TALOS-N software. The dashed line corresponds to the two standard deviations of a half-normal distribution, describing the CSPs. **j**, CSPD of amide groups calculated for monomeric and dimeric TrkB TMD. Red rectangles indicate the absence of data for a residue. Substantial differences in the effects are seen for S432-Y434 and V437-I439, consistent with LSD binding close to the N-terminus of the TrkB TMD.

Consistent with these observations, nuclear magnetic resonance (NMR) spectroscopy of isotope-labeled TrkB TMD incorporated into lipid bicelles detected chemical shift perturbations (CSPs) of amide groups of Y433, V437 and other residues within the TMD N-terminal side upon addition of LSD, which specifically take place in the dimeric state of the receptor (Fig. 2j,k and Extended Data Fig. 2). Together, these data suggest that the TrkB TMD dimer is a high-affinity primary target for psychedelics.

### TrkB binding site of psychedelics

**Extended Data Fig. 3:**
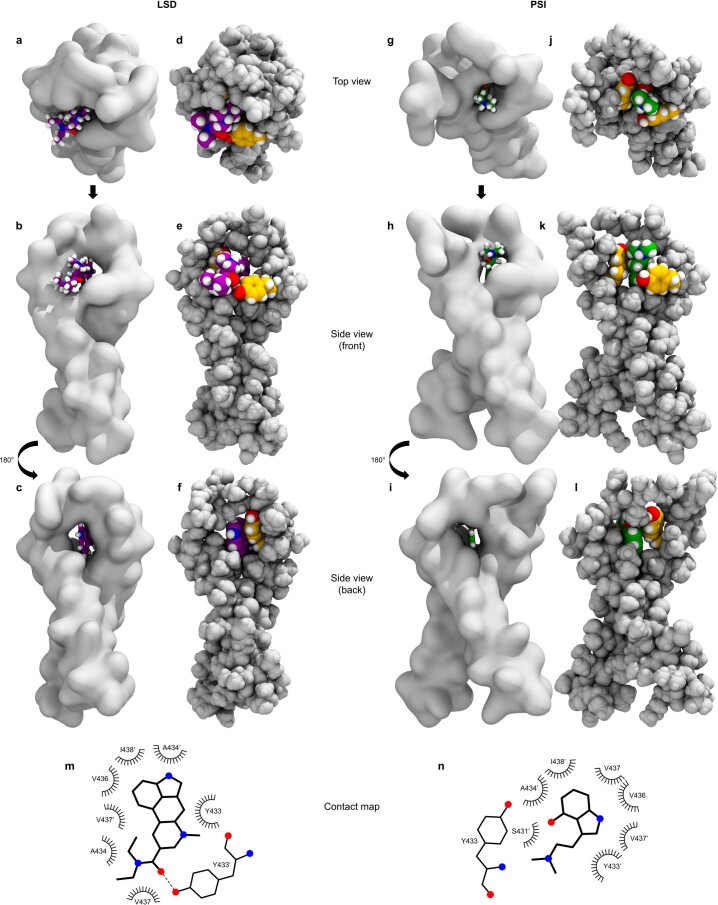
Representative atomistic MD snapshots showing the binding pocket for LSD^+^ (purple) and PSI^+^ (green) in the extracellular-facing crevice of the wild-type TrkB TMD dimer (gray). **a-c**, **g-i**, Drugs are shown in licorice representation and protein is shown in surface representation. **d-f**, **j-l**, Drugs and protein are shown in van der Waals representation. Y433 residues are shown with yellow carbon atoms. **m**, **n**, 2D diagrams of protein-ligand interaction for protonated LSD (**m**) and protonated PSI (**n**) in their binding pockets. Oxygen, nitrogen, and hydrogen atoms are shown in red, blue, and white, respectively.

**Extended Data Fig. 4:**
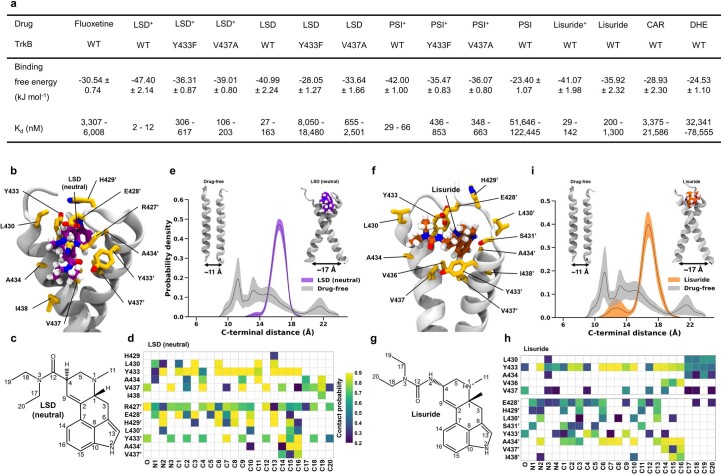
Modes and energetics of psychedelic binding to TrkB. **a**, Binding free energies (ΔG ± s.e.m.) and the ranges for the corresponding dissociation constants are given for the drugs (neutral and protonated forms) and the WT and mutant variants (Y433F, V437A) of TrkB TMD dimers. CAR: cabergoline; DHE: dihydroergotamine. **b**, Representative MD snapshot showing the binding pocket for neutral LSD (purple) in the extracellular-facing side of TMD dimer (gray). Side chains (yellow) of relevant binding site residues are displayed. **c, d**, Contact probability between the neutral LSD heavy atoms (**c**) and binding pocket residues (**d**). Residues of the second chain in the dimer are indicated with an apostrophe (‘). **e**, Distributions of TMD dimer C-terminal distance show that neutral LSD stabilizes the cross-shaped conformation of TrkB favorable for receptor activation in a 40 mol% CHOL membrane. Lines represent the mean distribution and bands represent the standard errors (n = 10 independent simulations). TMD conformations corresponding to indicated C-terminal distances and drug-bound states are shown in the inset. Representative MD snapshots are shown for TMD conformations corresponding to indicated C-terminal distances of drug-free and neutral LSD-bound states. **f**, Representative MD snapshot showing the binding pocket for lisuride^+^ (orange) in the extracellular-facing side of TrkB TMD dimer. **g, h**, Contact probability between lisuride^+^ heavy atoms (**g**) and binding pocket residues (**h**). **i**, Distributions of TMD dimer C-terminal distance show that lisuride^+^ stabilizes the cross-shaped conformation of TrkB favorable for receptor activation in a 40 mol% CHOL membrane. Lines represent the mean distribution and bands represent the standard errors (n = 10 independent simulations). TMD conformations corresponding to indicated C-terminal distances and drug-bound states are shown in the inset. Representative MD snapshots are shown for TMD conformations corresponding to indicated C-terminal distances of drug-free and lisuride^+^-bound states. Detailed statistics reported in Supplementary Table 1.

We used atomistic molecular dynamics (MD) simulations of the TrkB TMD dimers embedded in cholesterol (CHOL)-enriched membranes^11^ to identify the binding site of psychedelics. LSD and PSI spontaneously associate with TrkB TMD dimers. Consistent with our NMR structural data and mutagenesis binding studies, simulations revealed binding sites for LSD and PSI located in the extracellular-facing crevice of the crisscrossed TMD dimers such that both TMDs of a dimer are required for binding (Fig. 2a–c and Extended Data Fig. 3). Subsequent binding free energy estimations showed that LSD and PSI bind to the TMD of TrkB dimers with higher affinity than fluoxetine (Fig. 2d and Extended Data Fig. 4a). In agreement with our experimental data, Y433, A434 and V437 stabilize the binding of LSD and PSI (Fig. 1e–h and Extended Data Figs. 3 and 4b–d). Y433F and V437A mutations markedly decrease the binding affinity of psychedelics (Fig. 2d and Extended Data Fig. 4a). At the high CHOL concentration typical for synaptic membranes^16^, LSD stabilizes the TrkB TMD dimer in a conformation where the distance between its Cα residues (L451–L453) is ~17 Å (Fig. 2i and Extended Data Fig. 4e). Thus, LSD stabilizes TrkB dimers in a conformation that is more favorable to activation by BDNF, akin to the mechanism previously identified for fluoxetine^11^.

**Fig. 3:**
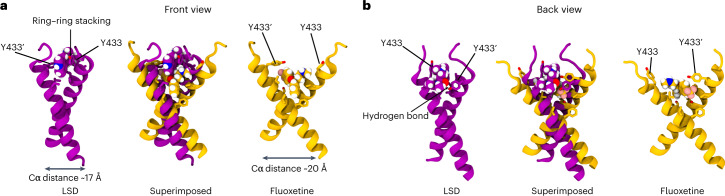
Different TrkB binding modes of LSD and fluoxetine. **a**,**b**, Representative snapshots of atomistic MD simulations showing the front (**a**) and back (**b**) views of the binding pockets for LSD (purple) and fluoxetine (yellow) in the extracellular-facing crevice of TrkB TMD dimers. Side chains of relevant binding site residues are displayed. Superimposed structures of TrkB optimally bound to LSD or fluoxetine reveal that, while some residues involved in binding are shared (Y433 and V437), the binding modes are different. Fluoxetine binds at a site deeper within the dimer, locking the TMD dimers in a more open cross-shaped conformation (distance between the center of mass L451–L453 Cα atoms of each monomer ~20 Å). In contrast, LSD binds closer to the N-terminus of the TrkB TMD and establishes more stable interactions with the dimer: a hydrogen bond between the oxygen atom of the diethylamide group of LSD and the Y433 residue of one monomer, and pi-stacking of the aromatic backbone of the drug with the Y433 residue of the second monomer, locking the TMD dimer in a tighter cross-shaped conformation (L451–L453 Cα distance ~17 Å) compared with fluoxetine. Drugs are shown in van der Waals representation. Oxygen, nitrogen and hydrogen atoms are shown in red, blue and white, respectively.

Notably, we found that the binding pockets for fluoxetine and LSD are distinct, even though their binding involves shared residues. Fluoxetine binds deeper within the TMD and requires membrane lipid stabilization^11^, locking the TMD dimer in a more open cross-shaped conformation (Cα distance ~20 Å). In contrast, LSD binds closer to the membrane surface and establishes more stable interactions with the dimer: a hydrogen bond between the oxygen of LSD’s diethylamide group and the Y433 residue of one monomer, and pi-stacking of the ergoline aromatic backbone with the Y433 residue of the second monomer, locking TMD dimers in a tighter cross-shaped conformation (Cα distance ~17 Å) compared with fluoxetine (Fig. 3). This is consistent with the deeper-located mutation S440A interfering with fluoxetine^11^ but not with LSD binding (Fig. 1a). PSI displays a binding pocket, interacting residues and stable drug:TrkB complex conformation (Cα distance ~17 Å) similar to that of LSD (Fig. 2a–i and Extended Data Fig. 3). Atomistic simulations and free energy calculations show that lisuride binds to TrkB with reduced affinity when compared with LSD (Extended Data Fig. 4a), but interacting residues and TMD dimer conformation (Cα distance ~17 Å) of lisuride are similar to those of LSD and PSI (Extended Data Fig. 4f–i). In contrast, control compounds cabergoline and dihydroergotamine display very poor TrkB binding (Extended Data Fig. 4a). Thus, psychedelics and antidepressants have distinct binding pockets and stabilize different conformations of the TMD dimer, which may underlie the disparities observed among TrkB ligand affinities.

### Psychedelics promote BDNF signaling

**Fig. 4:**
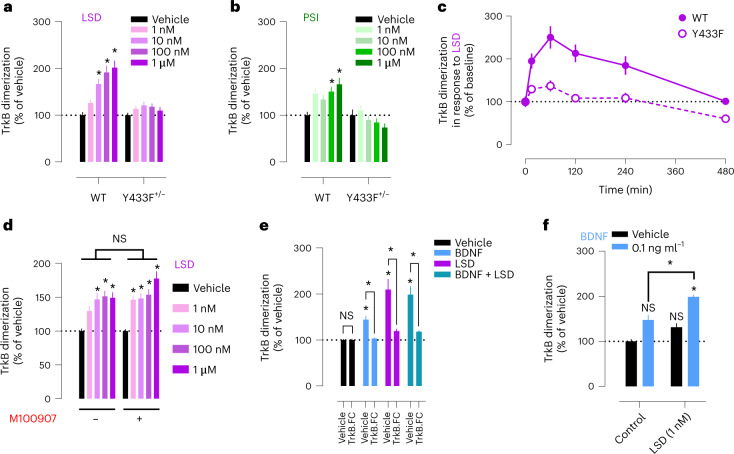
Psychedelics promote TrkB dimerization. **a**,**b**, LSD (**a**) and PSI (**b**) promote TrkB dimerization, but these effects are lost in Y433F^+/−^. **c**, Timeline of LSD-induced TrkB dimerization. **d**, M100907 does not prevent LSD-induced TrkB dimerization. **e**, TrkB receptor bodies (TrkB.FC) that hijack extracellular BDNF abolish the effects of both BDNF and LSD on dimerization. **f**, LSD potentiates the effects of low BDNF concentrations on TrkB dimerization. Data shown as mean ± s.e.m., *P < 0.05. NS, not significant. Detailed statistics reported in Supplementary Table 1.

**Extended Data Fig. 5:**
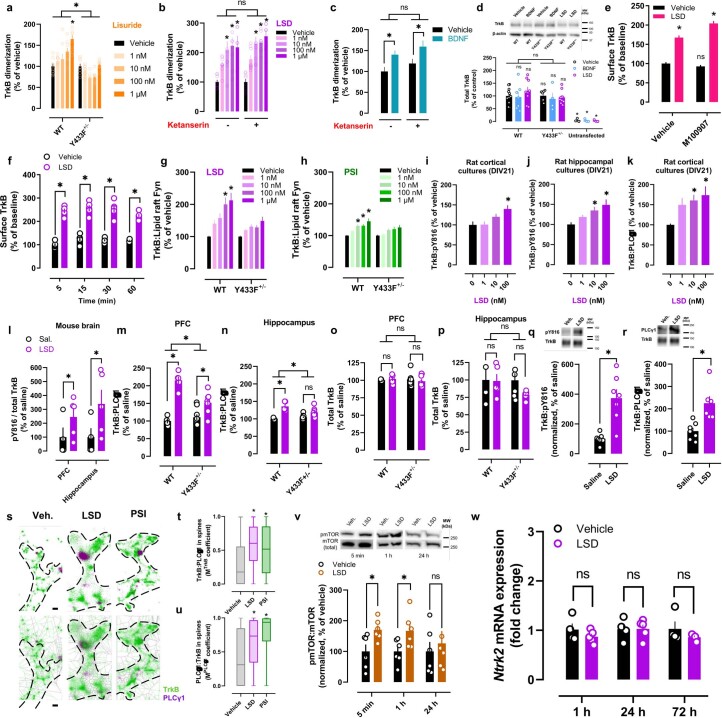
Psychedelics promote TrkB signaling. **a**, Lisuride promotes TrkB dimerization in a concentration-dependent manner, but these effects are lost in Y433F^+/−^ heterodimers. **b**, Ketanserin does not prevent LSD-induced TrkB dimerization. **c**, Ketanserin does not block the effects of BDNF on TrkB dimerization. **d**, No differences in TrkB protein expression between cells transfected to express TrkB WT homodimers or Y433F heterodimers, with or without BDNF and LSD treatments. **e**, LSD increases TrkB cell surface retention independently of 5-HT_2A_ activation. **f**, LSD rapidly increases TrkB retention in the cell surface, which is sustained for at least 1 h. **g, h**, LSD (**g**) and PSI (**h**) increase TrkB interaction with lipid raft-restricted Fyn, but this effect is lost in Y433F^+/−^ dimers. **i-k**, LSD increases TrkB phosphorylation (pY816) and TrkB:PLCγ1 interaction in rat cortical cultures (**i, k**), and pY816 in hippocampal cultures (**j**). **l**, A single injection of LSD increases pY816 in the PFC and hippocampus of mice. **m, n**, A single administration of LSD increases TrkB:PLCγ1 interaction in the PFC (**m**) and hippocampus (**n**), but these effects are impaired in Y433F^+/−^ mice. **o, p**, Total TrkB expression in PFC (**o**) or hippocampus (**p**) of WT or Y433F^+/−^ mice remain unaffected by a single dose of LSD. **q, r**, Immunoblot confirms that a single administration of LSD increases pY816 (**q**) and TrkB:PLCγ1 co-immunoprecipitation (**r**) in the PFC of mice. **s**, Representative single-molecule localization maps (top) or corresponding Voronoi diagrams (bottom) of dendritic spines after treatment with vehicle, LSD or PSI. TrkB in green, PLCγ1 in purple. Scale bar = 200 nm. **t, u**, SMLM demonstrate**s** that psychedelics increase colocalization of TrkB and PLCγ1 in dendritic spines of hippocampal neurons. Box-and-whisker plots (center line, median; box limits, upper and lower quartiles; whiskers, 1.5 interquartile range) show the distribution of Manders colocalization coefficients for TrkB:PLCγ1 (M^TrkB^) (**t**) and PLCγ1:TrkB (M^PLCγ1^) (**u**) per spine. **v**, Timeline of LSD-induced increase in pmTOR. **w**, *Ntrk2* mRNA expression shows no change after LSD treatment. Data shown as mean ± s.e.m., *P < 0.05. Detailed statistics reported in Supplementary Table 1.

We used a split-luciferase complementation assay to investigate TrkB dimerization and interactions in response to psychedelics^17^. LSD and PSI induce a fast and long-lasting increase in dimerization of wild-type (WT) TrkB at nanomolar concentrations, but this effect is abolished in WT:Y433F heterodimers (Y433F^+/−^) (Fig. 4a–c). Consistently, LSD increases phosphorylation of TrkB tyrosine 816 (pY816) residues in cortical and hippocampal cultures and in mouse brain samples after a single administration (Extended Data Fig. 5i,j,l,q), indicating facilitated dimerization. Lisuride similarly increases TrkB dimerization at higher concentrations (Extended Data Fig. 5a). Pretreatment with ketanserin or M100907 fails to prevent the effects of LSD and BDNF on TrkB dimerization in N2a cells that do not express 5-HT_2A_ messenger RNA (Fig. 4d, Extended Data Fig. 5b,c and Extended Data Table 1), indicating that psychedelics induce TrkB dimerization in the absence of 5-HT_2A_ and ketanserin and M100907 do not interfere with this effect.

TrkB.FC, which sequesters extracellular BDNF, prevents the effects of LSD and BDNF on TrkB dimerization (Fig. 4e). Furthermore, LSD potentiates the effects of very low concentrations of BDNF (0.1 ng ml^−1^) on TrkB dimerization (Fig. 4f), presumably by stabilizing TrkB dimerization. No differences in total TrkB expression were observed between WT and Y433F^+/−^ or following treatment with LSD or BDNF (Extended Data Fig. 5d). These data demonstrate that psychedelics do not directly activate TrkB, but their effects on TrkB dimerization are dependent on release of endogenous BDNF, consistent with an allosteric effect.

TrkB is mainly localized in intracellular vesicles and only transiently translocated to the cell surface where it is exposed to BDNF^18–20^. Psychedelics rapidly increase the neuronal surface retention of TrkB, independently from 5-HT_2A_ activation, and promote TrkB interaction with raft-restricted Src family kinase Fyn^21,22^, indicating increased localization to raft-like synaptic membranes. This effect is lost in Y433F^+/−^ heterodimers (Extended Data Fig. 5e–h).

**Fig. 5:**
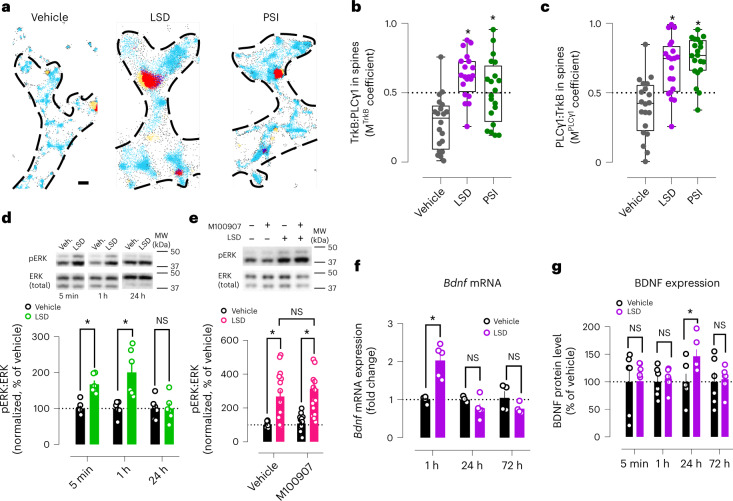
Psychedelics recruit neurotrophic signaling through BDNF and TrkB. **a**, Representative single-molecule localization microscopy maps of dendritic spines after treatment with vehicle, LSD and PSI. TrkB in light blue, PLCγ1 in yellow, TrkB:PLCγ1 in dark blue, PLCγ1:TrkB in red. Scale bar, 200 nm. **b**,**c**, Psychedelics increase colocalization of TrkB and PLCγ1 in dendritic spines. **d**, Timeline of LSD-induced increase in pERK. **e**, M100907 pretreatment does not prevent LSD-induced increase in pERK. **f**,**g**, Timelines of *Bdnf* mRNA (**f**) and BDNF protein expression (**g**) after LSD treatment. MW, molecular weight. Data shown as mean ± s.e.m. or box-and-whisker plots (center line, median; box limits, upper and lower quartiles; whiskers, maximum values; points, averaged Manders colocalization coefficient per neuron) (**b** and **c**), *P < 0.05. NS, not significant. Detailed statistics reported in Supplementary Table 1.

Psychedelics also promote BDNF downstream signaling. In neuronal cultures, LSD increases TrkB interaction with phospholipase C gamma 1 (PLCγ1) (Extended Data Fig. 5k), which docks on pY816 and regulates intracellular Ca^2+^ signaling and antidepressant action^11,23,24^. TrkB interaction with PLCγ1 is also increased in the prefrontal cortex (PFC) and hippocampus of WT but not Y433F^+/−^ mice, without affecting total TrkB expression (Extended Data Fig. 5m–p,r). Tracking single-molecule localizations of TrkB and PLCγ1 in neurons by direct stochastic optical reconstruction microscopy coupled with total internal reflection fluorescence (dSTORM/TIRF) confirmed that LSD and PSI increase TrkB:PLCγ1 colocalization in dendritic spines (Fig. 5a–c and Extended Data Fig. 5s–u). In addition, LSD rapidly increases phosphorylation of ERK (pERK), independently of 5-HT_2A_ activation (Fig. 5d,e), and promotes phosphorylation of mTOR for at least 1 h (Extended Data Fig. 5v). *Bdnf* mRNA is upregulated at 1 h after LSD treatment and BDNF protein levels are elevated at 24 h (Fig. 5f,g) without any changes in *Ntrk2* mRNA levels (Extended Data Fig. 5w). In summary, the effects of psychedelics on neurotrophic signaling are dependent on BDNF and TrkB, and do not require 5-HT_2A_ activation.

### Psychedelic-promoted plasticity depends on BDNF and TrkB

**Fig. 6:**
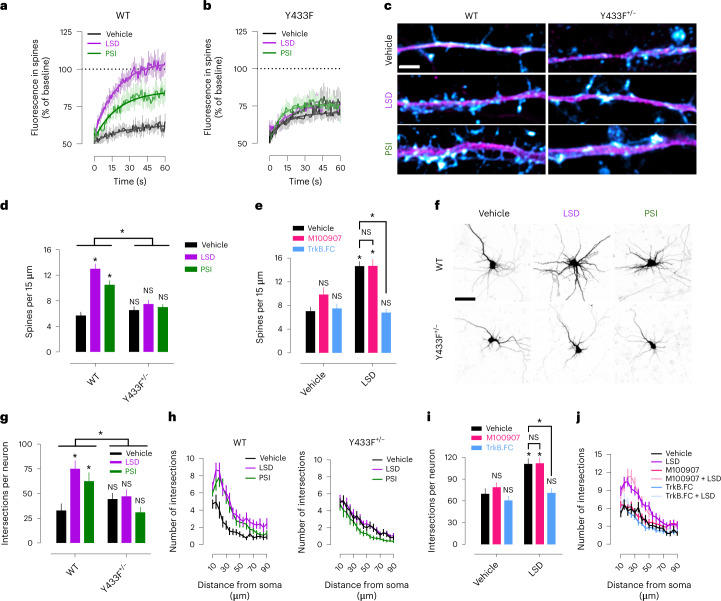
Psychedelic-induced neuroplasticity depends on TrkB and BDNF, but not 5-HT_2A_ activation. **a**,**b**, LSD and PSI produce rapid recovery of GFP-tagged TrkB fluorescence after photobleaching, indicating increased TrkB trafficking into dendritic spines (**a**). This effect is lost in GFP-tagged Y433F TrkB-expressing neurons (**b**). **c**, Representative images of spinogenesis experiments. MAP2 in magenta, phalloidin in cyan. Scale bar, 2 µm. **d**, LSD and PSI induce robust spinogenesis in mature neuronal cultures derived from WT but not Y433F^+/−^ mice after 24 h of LSD treatment. **e**, TrkB.FC but not M100907 prevents increase in spinogenesis produced by LSD. **f**, Representative images of dendritogenesis experiments. Scale bar, 50 µm. **g**,**h**, LSD and PSI enhance dendritic arbor complexity 72 h after treatment in neuronal cultures of WT but not Y433F^+/−^ mice as measured by Sholl analysis of neurite intersections. **i**,**j**, TrkB.FC but not M100907 prevents increase in dendritic arbor complexity produced by LSD. Data shown as mean ± s.e.m., *P < 0.05. NS, not significant. Detailed statistics reported in Supplementary Table 1.

**Extended Data Fig. 6:**
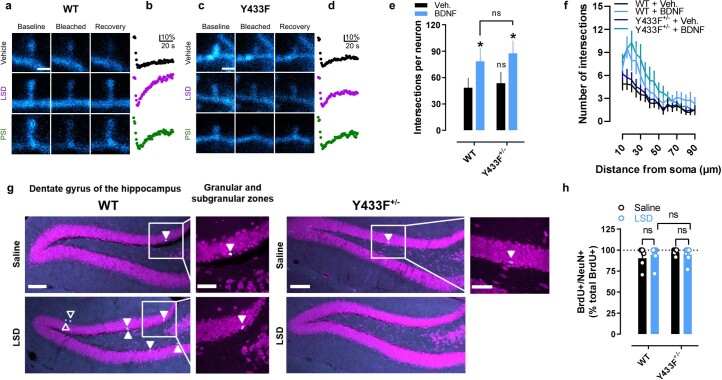
Effects of psychedelics on plasticity depend on TrkB. **a-d**, Psychedelics promote fast and robust localization of WT but not Y433F TrkB into dendritic spines of hippocampal neurons. Representative FRAP image series (**a**,**c**) and recovery traces (**b**,**d**) of data shown in Fig. 6a, b. Scale bar = 1 µm. **e,f**, BDNF increases dendritic arbor complexity in neuronal cultures of both WT and Y433F^+/-^ mice, indicating that the Y433F heterozygous mutation does not alter TrkB function and response to BDNF. **g**, Representative images of data shown in Fig. 7b from DGCs survival experiments with WT and Y433F^+/-^ animals treated with saline or LSD. BrdU (cyan), NeuN (magenta). Filled arrows indicate BrdU and NeuN nuclear colocalization. Scale bar = 100 µm. **h**, Percentage of mature DGCs (BrdU+/NeuN+) over total BrdU+ cells. Data shown as mean ± s.e.m., *P < 0.05. Detailed statistics reported in Supplementary Table 1.

We used fluorescence recovery after photobleaching (FRAP) in neuronal cultures expressing GFP-tagged TrkB to investigate the effects of psychedelics on TrkB trafficking. FRAP revealed that psychedelics induce rapid TrkB trafficking into dendritic spines. LSD robustly increases fluorescence recovery (+42.42% increment, *t*_1/2_ = 13.53 s), followed closely by PSI (+22.72% increment, *t*_1/2_ = 13.99 s), when compared with vehicle treatment (*E*_max_ = 62.88%, *t*_1/2_ = 16.93 s). However, LSD (+4.80% increment, *t*_1/2_ = 16.00 s) and PSI (+8.63% increment, *t*_1/2_ = 15.18 s) failed to increase fluorescence recovery of Y433F compared with vehicle treatment (*E*_max_ = 70.82%, *t*_1/2_ = 11.08 s) (Fig. 6a,b and Extended Data Fig. 6a–d).

Previous findings have reported that psychedelics increase neurite outgrowth and spine formation in cultured neurons^12,13^. Consistent with these findings, LSD and PSI increase spine density of mature neuronal cultures from WT mice at 24 h after treatment, but not of neuronal cultures from Y433F^+/−^ mice. Notably, TrkB.FC but not M100907 prevented the spinogenic effect of LSD (Fig. 6c–e), indicating that this effect is dependent on BDNF release but not on 5-HT_2A_ activation. Psychedelics also increase dendritic arbor complexity in neuronal cultures derived from WT but not Y433F^+/−^ mice. Again, TrkB.FC but not M100907 prevented LSD effects on dendritogenesis (Fig. 6f–j). Ketanserin has previously been reported to prevent the effects of LSD on dendritic arbor complexity in cultured neurons^12^; more research is needed to resolve this apparent discrepancy with present results, but the various off-target effects of ketanserin might at least partially explain this contradiction^25^. Importantly, Y433F^+/−^ neuronal cultures treated with BDNF showed increased dendritic arbor complexity and were indistinguishable from WT cultures (Extended Data Fig. 6e,f), indicating that the heterozygous Y433F^+/−^ mutation does not interfere with the TrkB response to BDNF. Furthermore, Y433F^+/−^ mice do not show any baseline differences from WT TrkB mice, and Y433F^+/−^ neuronal cultures and mice respond normally to BDNF in plasticity-related and behavioral assays^26^. These results indicate that Y433F^+/−^ does not compromise TrkB function or BDNF responses; it only abrogates the binding of psychedelics and antidepressants to TrkB and their effects on plasticity. Together these findings are consistent with previous reports of TrkB involvement in psychedelic drug-induced plasticity^9,12,13^, and support the notion that psychedelics are not direct TrkB agonists, but they facilitate the effects of endogenous BDNF, independently of 5-HT_2A_ activation.

### LSD effects on network plasticity and behavior

**Fig. 7:**
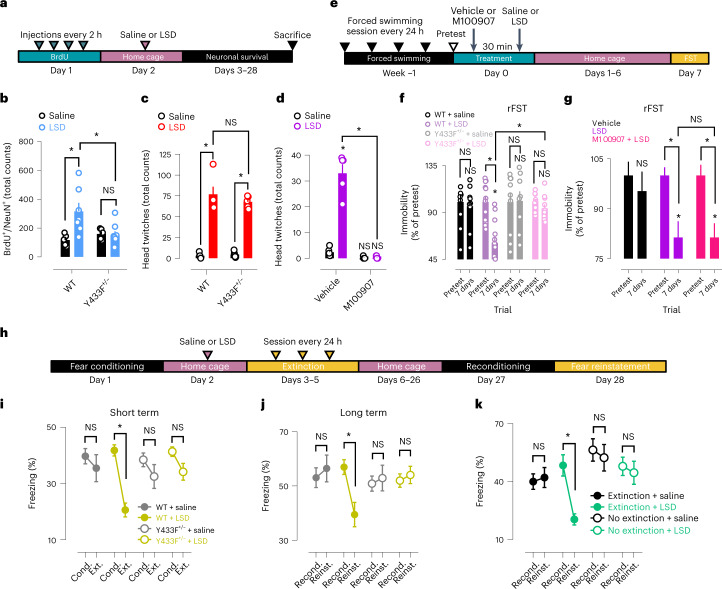
TrkB mediates plasticity-related and antidepressant-like effects of LSD on neuronal networks and behavior. **a**, Timeline of newborn DGCs long-term survival experiments. **b**, LSD increases survival of newborn DGCs of WT but not Y433F^+/−^ mice at 4 weeks after a single administration. **c**,**d**, Head twitches in response to LSD occur normally in Y433F^+/−^ mice (**c**) but are blocked by M100907 (**d**). **e**, Timeline of rFST experiments. **f**,**g**, A single dose of LSD produces a sustained antidepressant-like effect in rFST that is lost in Y433F^+/−^ mice (**f**) but is unaffected by M100907 pretreatment (**g**). **h**, Timeline of fear conditioning experiments. **i**,**j**, A single injection of LSD facilitates extinction training and reduces contextual freezing at 3 days (**i**) and also at 4 weeks (**j**) after administration in WT but not Y433F^+/−^ mice. **k**, LSD requires combination with extinction training to reduce contextual freezing in the long term. Locomotor activities at baseline and single traces of fear conditioning experiments shown in Extended Data Fig. 7e–s. Cond., conditioning; Ext., extinction; Recond., reconditioning; Reinst., reinstatement. Data shown as mean ± s.e.m., *P < 0.05. NS, not significant. Detailed statistics reported in Supplementary Table 1.

Chronic treatment with conventional antidepressants or a single administration of ketamine enhance neurogenesis and long-term neuronal survival of dentate granule cells (DGCs) of the hippocampus, and these effects are dependent on BDNF signaling through TrkB^27,28^. We found that LSD doubles the number of surviving DGCs of WT mice, but not of Y433F^+/−^ mice, at 4 weeks after a single administration (Fig. 7a,b and Extended Data Fig. 6g,h), indicating that the effects of LSD on DGC survival are mediated by their binding to TrkB.

**Extended Data Fig. 7:**
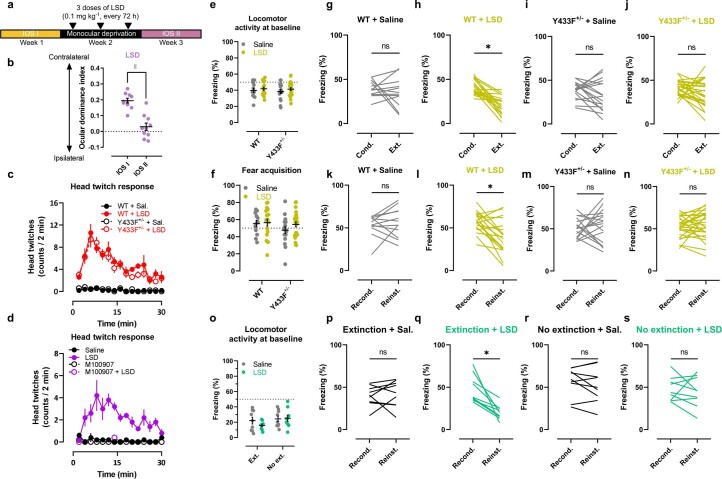
LSD effects on plasticity at the network and behavioral levels depend on TrkB. **a**, Timeline of ocular dominance plasticity experiment. **b**, LSD treatment combined with monocular deprivation induces an ocular dominance shift in the primary visual cortex of mice in favor of the opened eye. **c, d**, Time course of the head-twitch response immediately after LSD administration for data shown in Fig. 7c, d. **e-n**, Locomotor activity at baseline (**e**), fear acquisition response (**f**) and single traces of fear conditioning and extinction data shown in Fig. 7i, j after the administration of saline or LSD to WT and Y433F^+/-^ mice and their effects in the short-term (**g-j**) and long-term (**k-n**). **o-s**, Locomotor activity at baseline (**o**) and single traces (**p**-**s**) of fear conditioning data shown in Fig. 7k on administration of saline or LSD in combination with or without extinction training. Data shown as mean ± s.e.m, *P < 0.05. Detailed statistics reported in Supplementary Table 1.

We and others have previously shown that antidepressants^11,29^ and ketamine^11,30,31^ reactivate critical period-like plasticity in the adult visual cortex. We found that LSD also promotes visual plasticity and facilitates a shift in ocular dominance (OD) in favor of the open eye in the primary visual cortex of mice following a week of monocular deprivation (Extended Data Fig. 7a,b).

The head-twitch response has been used in rodents as a reporter of the hallucinogenic effects of psychedelics in humans and this response is known to be mediated by 5-HT_2A_ receptors^32^. We found that the head-twitch response occurs normally in Y433F^+/−^ mice treated with LSD, but is blocked by M100907 (Fig. 7c,d and Extended Data Fig. 7c,d). These results suggest that, as far as the head-twitch response reliably reports hallucinogenic actions of LSD in humans, these effects are dependent on 5-HT_2A_ activation but detached from TrkB activity, which indicates a dissociation between the hallucinogenic and plasticity-promoting effects of psychedelics.

In a rodent model of chronic stress consisting of repeated sessions of the forced swimming test (rFST)^33^, LSD produced a sustained antidepressant-like effect 7 days after a single administration in WT but not Y433F^+/−^ mice. Pretreatment with M100907 did not prevent the antidepressant-like effect of LSD (Fig. 7e–g). LSD also facilitates contextual extinction of conditioned fear response 72 h after a single administration, which persists for at least 4 weeks. However, these short- and long-term effects are lost in Y433F^+/−^ mice. Importantly, LSD alone does not bring about fear extinction, as extinction training is required to produce a sustained decrease in behavioral freezing after LSD treatment (Fig. 7h–k and Extended Data Fig. 7e–s). This is reminiscent of the effects of other antidepressants that depend on environmental input to exert their therapeutic-like effects^29,34,35^, and are consistent with the importance of ‘set and setting’ in modulating psychedelic effects in humans^36^. Taken together, these results strongly suggest that TrkB mediates the plasticity-related and antidepressant-like effects of LSD at the network and behavioral levels but is not involved in its hallucinogenic-like action.

## Discussion

Recent clinical studies suggesting that psychedelics produce rapid and long-lasting antidepressant effects^2^ have received tremendous attention. Mystical experiences induced by psychedelics have been associated with their clinical efficacy^37^. However, the hallucinogenic effects of psychedelics limit their widespread clinical application, as their administration is restricted to clinical settings that often require intensive monitoring^4,38^. A number of recent observations suggest that the antidepressant and plasticity-promoting effects of psychedelics may be dissociable from their hallucinogenic effects. First, plasticity-like effects of psilocybin are not blocked by the 5-HT_2A_ antagonist ketanserin in mice^8,9^, while it blocks the head-twitch response, a 5-HT_2A_-mediated behavior in rodents used as a proxy for hallucinogenic action of psychedelics in humans^32^. Second, derivatives of psychedelic compounds have recently been introduced that promote plasticity and antidepressant-like behavior but do not seem to produce head-twitch responses^7,39,40^. However, detailed information concerning the sites that mediate these differential actions have remained unclear.

**Extended Data Fig. 8:**
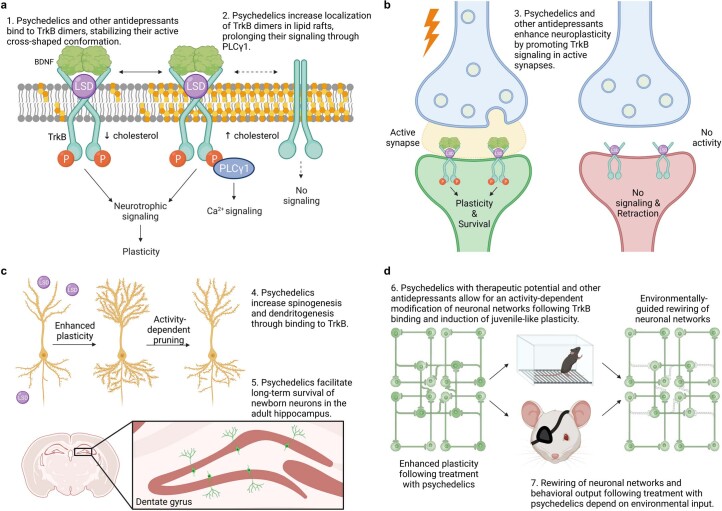
Working model. **a**, Psychedelics bind to the TMD of TrkB dimers, stabilizing their active cross-shaped conformation even at high-cholesterol concentrations characteristic of synaptic membranes, where dimers would normally adopt their inactive parallel conformation^11^. Psychedelics rapidly facilitate BDNF signaling and induction of plasticity by promoting TrkB dimerization, cell surface retention and localization into lipid rafts. There, TrkB interacts with Src kinases like Fyn and recruits PLCγ1, ERK and mTOR neurotrophic signaling pathways. **b**, Psychedelics promote synaptic plasticity in an activity-dependent manner. Activity increases endogenous BDNF release and psychedelics act as positive allosteric modulators facilitating TrkB neurotrophic signaling. This results in dendritic spines being formed or reinforced, while inactive spines are retracted. **c**, Psychedelics increase spinogenesis and dendritogenesis following TrkB activation, increasing the pool of available synapses. Neuronal networks are rewired following activity-dependent synaptic pruning. Psychedelics also increase survival of DGCs in the hippocampus, which can be incorporated into the dentate gyrus local circuitry after maturation. **d**, As psychedelics readily penetrate the brain and TrkB is widely expressed across many brain areas and cell-types, the enhanced state of plasticity induced by psychedelics and other antidepressants that bind to TrkB may allow for a relatively widespread rewiring of neuronal networks (for example PFC, visual cortex, hippocampus). However, the enduring changes at the network and behavioral levels following psychedelic-induced plasticity depend on environmental input (for example extinction training, monocular deprivation). Thus, the therapeutic potential of psychedelics for treating psychiatric disorders may lie in their combination with environmental support, and not in their induction of plasticity alone. Network representations in this panel were modified from Castrén, E.^93^.

Here we show that psychedelics promote neuroplasticity and plasticity-related behavioral effects through their high-affinity binding to TrkB (Extended Data Fig. 8). Psychedelics are not direct TrkB agonists, as extracellular BDNF is necessary for their effects on TrkB dimerization and plasticity, but they act allosterically by facilitating the effects of endogenous BDNF released in active synapses, similar to what we recently found for other antidepressants^11^. Activity-dependent release of BDNF in stimulated synapses helps to selectively stabilize active synapses at the expense of inactive ones^41^, which is critical for Hebbian-type plasticity. Direct TrkB agonists are expected to indiscriminately activate TrkB in active and inactive synapses, which leads to gradual reduction of signal-to-noise ratio within neuronal networks. Therefore, through a positive allosteric modulation of BDNF signaling, psychedelics selectively promote, maintain and strengthen activity-dependent plasticity in active synapses.

A point mutation in the critical Y433 residue that impairs psychedelics binding to TrkB also abolishes induction of neuroplasticity and long-term plastic responses, but does not affect the head-twitch response. Moreover, 5-HT_2A_ antagonists fail to prevent psychedelic-induced TrkB dimerization and neurotrophic signaling, spinogenesis, dendritogenesis and antidepressant-like behavioral effects. These findings are perhaps not completely surprising, as there is still some debate in the field about whether 5-HT_2A_ receptors mediate the therapeutic effects of psychedelics. Overall, our results suggest that the TrkB-dependent effects of psychedelics on plasticity can be detached from their hallucinogenic-like effects mediated by 5-HT_2A_. We further show that lisuride also binds to TrkB, albeit with a lower affinity than LSD and PSI, which is consistent with recent evidence suggesting that lisuride increases dendritogenesis and spinogenesis in culture neurons^42^.

We have recently found that a dimer of TrkB TMDs contains a binding site for antidepressants, and that their plasticity-promoting and antidepressant-like effects are dependent on this interaction^11^. However, the affinity of conventional antidepressants to TrkB, albeit pharmacologically relevant, is low. Brain concentrations required for selective serotonin reuptake inhibitors binding to TrkB are achieved only after long-term treatment, which may partially explain the delay in the onset of their antidepressant action. Psychedelics readily penetrate into the brain after a single dose and, as shown here, bind to TrkB with much higher affinities than antidepressants in current clinical use. This may contribute to the fast and potent induction of neuroplasticity and more persistent behavioral effects produced by psychedelics when compared with other antidepressants^43^.

We have here characterized at an atomistic level of detail the binding sites of LSD, PSI and lisuride at TrkB TMD dimers, and used several control compounds to define binding specificity. We have also described the TrkB TMD conformational changes induced by psychedelics. Notably, the TMD binding pockets and TrkB conformational changes induced by antidepressants and psychedelics are distinct. However, it is clear that these compounds have different polypharmacological profiles beyond TrkB and 5-HT_2A_ that probably contribute to the overall effects and timeframes of each particular drug.

In conclusion, our findings support TrkB as the key target for psychedelic drug-induced plasticity. These data confirm TrkB as a common binding target for antidepressants and open an avenue for structure-based design of high-affinity TrkB-selective ligands with fast and long-lasting antidepressant action, but potentially devoid of hallucinogenic-like activity.

## Methods

### Animals

Female and male C57BL/6NTac-NTrk2em6006 (Y433F^+/−^) mice were generated and genotyped as previously described^11^, and their WT littermates were used as controls. Female and male C57BL/6JRccHsd WT mice (Envigo) were also used where indicated. All animals were 17–19 weeks old at the beginning of experiments, unless indicated otherwise. Animals were housed up to four mice per cage (Tecniplast, type-II individually ventilated cages GM500, 391 × 199 × 160 mm), except during behavioral testing or if single-cage separation was necessary to avoid fighting. They were kept under a 12 h light/dark cycle (lights on at 7:00), with free access to water and food. Behavioral experiments were performed during the light cycle. Previous studies were employed to estimate the sample size required for animal experiments. Animals were randomly assigned to experimental groups, and behavioral testing was conducted by experimenters blinded to genotype/treatment conditions. All animal experiments were performed in compliance with institutional guidelines and approved by the Regional State Administrative Agency for Southern Finland (ESAVI/38503/2019).

### Cell cultures

HEK293T cells transiently expressing GFP-tagged TrkB (WT, Y433F, V437A and TrkA.TM) were used for binding assays and MST, and N2a cells transiently expressing phGLuc-TrkB (WT and Y433F) and phGLuc-LR/Fyn were used for protein-fragment complementation assay (PCA). Cell lines were cultured and maintained as previously described^11^. Cortical and hippocampal primary neuronal cultures derived from rat or mouse embryos (embryonic day 18, E18) were dissected, plated and maintained as previously described^44^. Experiments were performed with mature neuronal cultures (21 days in vitro, DIV21), unless otherwise specified. Lipofectamine 2000 (Invitrogen) was used for transfection of cell lines and primary neuronal cultures.

### Drugs

Ketamine hydrochloride (ketamine, Tocris, 3131), R,R-HNK (Tocris, 6094), (+)-LSD (Sigma-Aldrich, L7007), PSI (Biosynth Carbosynth, P-7800), lisuride maleate (Tocris, 4052), cabergoline (Sigma-Aldrich, C0246), ketanserin (+)-tartrate (ketanserin, Sigma-Aldrich, S006) and M100907 (Sigma-Aldrich, M3324) were solubilized in dimethyl sulfoxide (DMSO) and diluted 1:1,000 (final concentration 0.1% DMSO) for in vitro experiments. Drugs were diluted in sterile 0.9% saline and injected intraperitoneally (10 ml kg^−1^) for in vivo experiments. All controlled psychotropic substances were obtained, stored, handled and disposed of as regulated under the permit issued by the Finnish Medicines Agency to the Faculty of Medicine, Medicum (FIMEA/2022/000945).

### Ligand binding assays

Binding assays were performed as previously described with minor modifications^11^. Briefly, HEK293T cells transiently expressing TrkB (WT, Y433F, V437A, S440A or TrkA.TM chimera) were lysed (20 mM Tris–HCl, 137 mM NaCl, 10% glycerol, 50 mM NaF, 1% Nonidet P-40, 0.05 mM Na_3_VO_4_, containing protease inhibitor (Sigma-Aldrich, P2714) and phosphatase inhibitor (Sigma-Aldrich, P0044) cocktails), vortexed, sonicated and centrifuged 24 h after transfection, and 150 µg of total protein per sample was loaded onto 96-well plates (PerkinElmer, OptiPlate 96F-HB) that had been precoated with an anti-TrkB antibody (R&D Systems, AF1494; 1:500, overnight, 4 °C) in carbonate buffer (pH 9.8) and blocked with 3% bovine serum albumin (BSA) in phosphate-buffered saline (PBS) for 2 h at room temperature. The use and validation of the anti-TrkB antibody in these binding assays has been previously described in the literature^11^. Samples from mouse embryo (E18) brains were also lysed, vortexed, sonicated and centrifuged for binding assays. Plates were then washed with PBS. For radioligand saturation binding assays, we incubated ^3^H-LSD (PerkinElmer, NET638250UC) in PBS at 1 pM to 100 nM final concentrations for 1 h at room temperature to determine the *K*_d_ and *B*_max_. Cold LSD at 10 µM final concentration was used to determine nonspecific binding in wells with matching concentrations of the radioligand. For radioligand competition binding assays, 10 nM final concentration of ^3^H-LSD was incubated with 1 nM to 10 µM of drugs in PBS for 2 h at room temperature to calculate the *K*_i_ values. Nonspecific binding was determined with wells containing the same concentration of radioligand with 10 µM of cold LSD. For radioligand dissociation binding assays, 10 nM final concentration of ^3^H-LSD in PBS (2% BSA) was pre-incubated for at least 2 h at room temperature before the addition of excess cold LSD (500 nM final concentration) at the designated timepoints (2 min, 15 min, 30 min, 1 h, 2 h, 4 h and 8 h) and finally washed with PBS. Radioactive emission of bound ^3^H-LSD for saturation, competition and dissociation binding assays was determined by scintillation (PerkinElmer, OptiPhase SuperMix, 1200-437). For the competition binding assays with biotinylated R,R-HNK, the amino-biotinylation of R,R-HNK was performed using a commercial kit (Thermo Scientific, EZ-Link NHS-PEG4 Biotinylation Kit, 21455) and the reaction monitored by MS. A mixture of biotinylated R,R-HNK (10 µM) and unlabeled compounds (1 pM to 10 µM) in PBS was added to the 96-well plates containing the immunoprecipitated TrkB and incubated for 1 h at room temperature. For biotin-labeled BDNF (bBDNF, Alomone Labs, #B-250-B) assays, the immunoprecipitated TrkB was incubated with bBDNF (0.01–10 ng ml^−1^) for 1 h. Luminescence was determined via streptavidin conjugated with horseradish peroxidase (HRP; Thermo Fisher Scientific, 21126; 1:5,000, 1 h at room temperature) in reaction with the Pierce ECL substrate (Thermo Fisher Scientific, 32106). Luminescence from blank wells that contained all reagents but the sample lysates was used to determine nonspecific binding. To obtain the binding curves, results from binding assays were fitted to the equation of specific binding with Hill slope from GraphPad Prism 9.2. To calculate *k*_off_ values, results were fitted to the equation of dissociation of one phase exponential decay from GraphPad Prism 9.2.

### MST

MST experiments were performed using the Monolith NT.115 (Blue/Red) instrument (NanoTemper Technologies). Lysates of HEK293T cells expressing GFP-tagged TrkB (WT, Y433F, TrkA.TM) were lysed, vortexed, sonicated and centrifuged 24 h after transient transfection and used as a source of fluorescently labeled TrkB, similarly to previously described protocols^11,45–47^. The advantage of this approach is the possibility of studying the proteins in close-to-native conditions. However, the presence of other proteins, lipids, nucleic acids and other components of crude cell lysates can alter the diffusion of biomolecules and therefore binding affinities. High viscosity and background signal typically results in the affinity values being orders of magnitude lower than those of more traditional binding assays. To evaluate binding of LSD, PSI, lisuride and BDNF (Peprotech, 450-02) to TrkB, cell lysates were diluted 1:2 with MST buffer (10 mM Na-phosphate buffer, pH 7.4, 1 mM MgCl_2_, 3 mM KCl, 150 mM NaCl and 0.05% Tween-20) to provide optimal level of initial fluorescence. The lysates of nontransfected HEK293T cells were used to evaluate background fluorescence, which was undetectable in the MST setup used in the present study. Titration series of LSD, PSI, lisuride or BDNF were mixed 1:1 and incubated with diluted cell lysates. The measurements were performed in premium coated capillaries (NanoTemper Technologies, MO-K025) using a LED source with 470 nm and 50% infrared-laser power at 25 °C. Analysis was performed using MO Affinity Analysis 2.3. Results were fitted to specific binding with Hill slope equations from GraphPad Prism 9.2.

### NMR spectroscopy

1,2-dimyristoyl-sn-glycero-3-phosphocholine (DMPC) and 1,2-dihexanoyl-sn-glycero-3-phosphocholine (DHPC) were purchased from Avanti Polar Lipids. Trifluoroethanol (TFE) was provided by Alfa Aesar. Deuterium oxide and deuterated TFE were products of the Cambridge Isotope Laboratories. SDS, Tris, tris(2-carboxyethyl)phosphine (TCEP) and ethylenediaminetetraacetic acid were purchased from Sigma-Aldrich.

The gene encoding transmembrane residues of human TrkB (TrkB TMD, T423-G466, UniProt ID Q16620) was amplified by PCR based on the TrkB gene (Cloning Facility). The PCR product was treated with endonucleases NdeI and HindIII and was cloned into the pGEMEX1 vector. ^15^N- and ^15^N-^13^C-labeled TrkB TMD was produced using the cell-free continuous exchange expression system based on the S30 *Escherichia coli* extract as described earlier^48^ TrkB TMD was purified using the size-exclusion chromatography. Precipitate from 3 ml of cell-free reaction mixture was washed two times with aqueous buffer (20 mM Tris, pH 8.0, and 150 mM NaCl) and solubilized with 500 μl of buffer (20 mM Tris, pH 8.0, 150 mM NaCl, 1.5% lauryl sarcosine, 10 mM 2-mercaptoethanol and 1 mM ethylenediaminetetraacetic acid). After centrifugation (60 min at 25,000*g* at room temperature) the clarified protein solution was applied onto a 10/600 Tricorn column packed with Superdex 200 prep grade (GE Healthcare) and pre-equilibrated with SEC buffer (20 mM Tris, pH 8.0, 50 mM NaCl, 0.5% lauryl sarcosine and 5 mM 2-mercaptoethanol). Protein-containing fractions were combined and precipitated by a trichloroacetic acid/acetone procedure^49^

^15^N- and ^15^N-^13^C-labeled TrkB TMD as a dry precipitate was dissolved in 450 µl of TFE/water 2:1 mixture. Then DMPC, 3-[(3-cholamidopropyl)dimethylammonio]-1-propanesulfonate, TCEP and water were added up to TFE/water vol/vol ratio of 1:1 with subsequent freeze-drying and resuspension in 450 μl of an aqueous buffer (20 mM NaPi, 1 mM TCEP and 0.01% vol/vol NaN_3_, pH 6.0) for ^15^N-labeled samples and 330 μl for the ^15^N-^13^C-labeled one. Samples for the chemical shift assignment contained 1.1 mM ^15^N-^13^C-labeled TrkB TMD, 9.6 mM DMPC and 42.7 mM DHPC. The effective DMPC/DHPC ratio was equal to 0.3, taking into account the concentration of monomeric DHPC in solution, determined using our previously published approach based on the NMR diffusion measurement^50^. Lipid-to-protein ratio was equal to 40. Samples for the LSD titration contained 0.2 mM ^15^N-labeled TrkB TMD, 3.6 mM DMPC and 21.8 mM DHPC, resulting in the effective DMPC/DHPC ratio of 0.3 as well. Lipid-to-protein ratio was equal to 80 to ensure the presence of both the dimeric and monomeric state of the protein in NMR spectra.

NMR spectra for the chemical shift assignment of TrkB TMD were recorded at 40 °C using the 600 MHz Bruker Avance III NMR spectrometer equipped with a triple-resonance cryogenic probe. Chemical shift assignment of the TrkB TMD backbone resonances, highlighted in the two-dimensional (2D) ^1^H,^15^N-TROSY-HSQC spectrum (Extended Data Fig. 2a), was accomplished with three-dimensional (3D) HNCO, HN(CO)CA, HNCA and ^1^H,^15^N-NOESY-HSQC spectra. Protein side chains were assigned using 3D ^1^H,^13^C-NOESY-HSQC and HCCH-TOCSY experiments. The obtained chemical shifts were processed using the TALOS-N software to determine the secondary structure of the peptide^51^. Initial analysis of NMR spectra revealed that the protein adopts the alpha-helical conformation in the region spanning from H430 to H459 (Extended Data Fig. 2b) and may reside in two distinct states, which is manifested by the presence of two sets of signals in HSQC NMR spectra. Populations of states were depending on the lipid-to-protein ratio (Extended Data Fig. 2c), which corresponds to the monomer-dimer transitions, and the state, which is abundant at high excess of lipids, was assigned to the monomeric form of TrkB TMD.

To analyze the interaction between the TrkB TMD and LSD, 0.2 mM samples of ^15^N-labeled TrkB TMD in DMPC/DHPC bicelles were titrated with the drug as follows. Lyophilized protein was resuspended using 95% H_2_O:5% D_2_O. LSD was first solubilized in acetonitrile at a concentration of 1 mg ml−1. Then LSD solution was aliquoted to have in the final sample LSD:TrkB TMD ratios of 0.25, 0.5, 1, 2, 4, 8 and 17.6. The aliquots were vacuum-dried using Eppendorf Concentrator Plus in 1.5 ml tubes and stored in the dark at −20 °C until usage. The titration was carried out using 5 mm tubes in the 850 MHz Bruker Avance III HD spectrometer equipped with a cryogenically cooled ^1^H, ^13^C, ^15^N triple-resonance probe head, using 450 μl of TrkB TMD in DMPC/DHPC bicelles sample. First, a set of reference spectra, including one-dimensional (1D) ^1^H as well as 2D ^1^H,^15^N-TROSY-HSQC for the ^15^N TrkB TMD protein without the ligand were measured. Then the sample was taken from the NMR tube and mixed with the dry aliquot of LSD pre-equilibrated at room temperature for 30 min. The solution was gently mixed by pipetting and centrifuged at 17,000*g* for 10 s and placed back in the NMR tube. The sample preparation was the same for each LSD concentration point, and the same set of spectra was collected as described above. The increasing amount of LSD in the sample was additionally controlled by following a well-separated NH resonance from the fused indole ring at δ_H_ 10.15 p.p.m. using 1D NMR. All the samples contained 0.25 mM of TSP-d4 as an internal chemical shift reference standard.

The obtained data were processed using the CSP approach. Chemical shifts of the individual signals were tracked in ^1^H,^15^N-TROSY-HSQC spectra, and the perturbations of peak positions in both dimensions of NMR spectra were combined to obtain the generalized chemical shift change:

1 $${\mathrm{CSP}} = \sqrt {\Delta \delta _{\mathrm{H}}^2 + \frac{{\Delta \delta _{\mathrm{N}}^2}}{{100}}}$$

where $\Delta \delta _{\mathrm{H}}^2$ and $\Delta \delta _{\mathrm{N}}^2$ are the chemical shift changes of protons and nitrogens, respectively, while 100 is introduced to take into account the difference in gyromagnetic ratios of these two nuclei.

The resultant CSPs for all TrkB TMD residues were then processed using the previously reported approach^52^. In brief, CSPs were approximated by a half-normal distribution, and residues with CSPs exceeding two standard deviations were considered as outliers (Extended Data Fig. 2g–i). To take into account the nonspecific chemical shift changes, most likely occurring due to the drug-induced variation in the properties of the lipid environment, we used the CSP difference approach. Since LSD and citalopram provide different magnitude of CSPs, we took the titration points with the closest average value of CSPs and measured the distance between the peak positions in the NMR spectra of TrkB TMD samples with LSD and citalopram using equation (1), where $\Delta \delta _{\mathrm{H}}^2$ and $\Delta \delta _{\mathrm{N}}^2$ are the chemical shift changes of protons and nitrogens between these two samples (Extended Data Fig. 2j). Such an approach allows the contrasting of chemical shift changes that are specific for LSD. As a result, we observed that substantial differences in the effects of the two drugs are seen only for S432-Y434 and V437-I439, and the CSPs of the rest of residues seem to be nonspecific due to alteration in lipid packing and/or effective lipid-to-detergent ratio caused by the penetration of the drug into bicelles.

### Liquid chromatography coupled to tandem MS of TrkB-associated proteins

Proteins associated with TrkB in HEK293T transfected cells were determined by MS following immunoprecipitation of TrkB. For immunoprecipitation, the raw lysate of HEK293T cells transiently expressing TrkB (WT, Y433F and V437A) were incubated overnight (4 °C, under light rotation) with anti-TrkB (R&D systems, AF1494) at 1 µg of antibody per 500 µg of total protein. Following incubation with 30 μl of Protein G-Sepharose (Life Technologies, 101242), the samples were centrifuged and the supernatant discarded. The precipitate was washed six times with Tris-buffered saline (TBS) and stored at −80 °C. Samples were then washed several times with a detergent-free buffer, and bound proteins were eluted with urea buffer (8 M urea in 50 mM ammonium bicarbonate). Proteins were reduced with TCEP (Sigma-Aldrich, C4706), alkylated with iodoacetamide (Sigma-Aldrich) and digested with Sequencing Grade Modified Trypsin (Promega, V5111), after which the samples were desalted using C18 microspin columns (Nest Group).

Liquid chromatography coupled to tandem MS analysis was done with a Q Exactive ESI-quadrupole-orbitrap mass spectrometer coupled to an EASY-nLC 1000 nanoflow LC (Thermo Fisher Scientific), using the Xcalibur version 3.1.66.10 (Thermo Scientific). The peptides were separated by a 60 min linear gradient using the C18-packed analytical column (Acclaim PepMap 100, 75 μm × 15 cm, 2 μm, 100 Å). The MS analysis was performed as data dependent acquisition in positive-ion mode. MS spectra were acquired from *m*/*z* 200 to *m*/*z* 2000 with a resolution of 70,000. The ten most abundant ions with charge states from 2+ to 7+ were selected for subsequent fragmentation (higher-energy collisional dissociation), and tandem MS spectra were acquired with a resolution of 17,500. Dynamic exclusion duration was 30 s. For each sample three biological replicates were analyzed. The raw MS data were processed with Andromeda search engine^53^ via Maxquant version 1.6.0.16 (ref. ^54^). Searches were done against the reviewed human Uniprot database (release 03/2020) supplemented with porcine trypsin and green fluorescence protein sequences (total of 20,303 entries). Carbamidomethylation (+57.021 Da) of cysteine residues was used as static modification and oxidation (+15.994 Da) of methionine was used as dynamic modification. Precursor mass tolerance and fragment mass tolerance were set to less than 20 p.p.m. and 0.1 Da, respectively. A maximum of two missed cleavages was allowed, and the results were filtered to a maximum false discovery rate of 1%. Contaminant Repository for Affinity Purification database (CRAPome, www.crapome.org/) were used to identify high-confidence interactors on the basis of the tandem MS counts.

### Atomistic MD simulations

The TM dimer of TrkB (residues 427–459) was modeled as previously described^11^. An extensive set of atomistic MD simulation systems (Supplementary Table 3) was prepared on the basis of this model to (1) explore the LSD binding site and mode, (2) optimize the binding modes of each drug (LSD, LSD^+^, PSI, PSI^+^, lisuride^+^, lisuride, cabergoline and dihydroergotamine) in the WT and mutant (Y433F and V437A) dimers, (3) estimate the drug binding affinities and (4) explore the effects of CHOL and the drugs on the conformation of the protein. The binding mode and site for neutral LSD was characterized using 100 independent simulations, each 1 μs long (system 1). In each of these simulation repeats, starting with the TrkB dimer embedded in a membrane (80:20 mol:mol POPC:CHOL), LSD was initially placed within the vicinity of the extracellular crevice in a different random orientation. The simulation data were then analyzed for stable association of the drug. The identified binding mode and site for neutral LSD was later used as an initial estimation for LSD^+^, PSI, PSI^+^, lisuride^+^, lisuride, cabergoline and dihydroergotamine in systems 2–9, where the binding site for each drug was optimized in separate 2-μs-long simulations. For both psychedelics, the predominant titration state in an aqueous solution at neutral pH is the protonated form (p*K*_a_ > 7.8) (ref. ^55^). LSD and PSI display higher affinity for TrkB in protonated form compared with their neutral form. The drug binding modes were further optimized for selected homozygous mutants (Y433F and V437A) in systems 10–15. The binding affinities for each drug for the respective optimized binding mode were estimated using the free energy perturbation (FEP) method with 24 windows (60 ns each) coupled via the Hamiltonian replica exchange scheme (FEP/HREX, Systems 16–29). To explore the effect of CHOL and the drugs on the conformation of the protein, additional systems were prepared by re-embedding the drug-bound proteins in 60:40 (mol:mol) POPC:CHOL membrane and/or also removing the bound drug (systems 30–34). Ten repeats were performed, each initiated with different velocities. For neutral PSI, only optimization of the binding mode and binding affinity estimation for the WT protein were performed, which showed low binding affinity.

All membrane–protein systems were constructed using CHARMM-GUI^56^ using the Charmm36(m) force field for the protein^57^ and lipids^58^, the TIP3P model for water^59^, and a compatible parameter set for ions^60^. All drugs were parametrized using the CHARMM-GUI ligand reader and modeler^61^ with the CHARMM general force field (CGenFF)^62^. The systems were solvated and neutralized with 150 mM KCl solution. The equilibration protocol with subsequent simulations in the canonical (NVT) and isothermal-isobaric (NPT) ensembles as suggested by CHARMM-GUI were performed before the production runs. All production runs were performed using GROMACS 2020.6 (ref. ^63^) at 310 K and 1 bar pressure with the same parameters given in previous studies^11^. FEP/HREX simulations^64^ were also performed as previously described for in silico free energy estimations^11^. The binding free energies and their errors were estimated using the multistate Bennet acceptance ratio method^65^ as implemented in gmx bar and discarding 4–20 ns from the beginning of each window.

The distributions of the distance between the center of mass of L451–L453 Cα atoms at the C-terminus of the TM dimer shown in Fig. 2i were computed as an average over the separate kernel density estimates performed using the last 200 ns and a sampling interval of 10 ns (200 samples) for each of ten independent simulations. The contact probability maps shown in Fig. 2g,h were calculated using a cutoff of 5 Å between the heavy atoms from the fully coupled state of the FEP/HREX simulations after discarding the first 20 ns. The total timescale of atomistic MD simulations was about 730 μs.

Marvin^66^ was used for drawing 2D chemical structures, LigPlot+ (ref. ^67^) for creating 2D diagrams of protein–ligand interaction, and VMD^68^ for creating 3D representations. The putative structural model of full-length TrkB dimer (Fig. 2b) was built on the basis of the structures of PDB IDs 1hcf (ref. ^69^), 2ifg (ref. ^70^) and 4asz (ref. ^71^). Matplotlib^72^ was used to create plots and SciPy^73^ for statistical analyses.

### PCA

PCA was used as a protein–protein interaction assay in live N2a cells to evaluate TrkB (WT and Y433F) dimerization and interaction with the lipid raft reporter protein cleaved Fyn (ref. ^22^) in response to treatment with LSD, PSI or lisuride (1 nM to 1 µM). PCA was also used to assess the effects of pretreatment with ketanserin (100 nM), M100907 (100 nM) or TRKB.FC (200 ng ml^−1^, R&D Systems, 688-TK-100) (ref. ^74^) on TrkB dimerization induced by LSD or BDNF (0.1 ng ml^−1^ or 10 ng ml^−1^). Ketanserin, M100907 and TRKB.FC were added to the media 10 min before treatment. The PCA method was performed as previously described^11,75,76^. Split humanized *Gaussia princeps* luciferase (hGLuc) was cloned on the C-terminus of the reporter proteins TrkB (WT and Y433F) and lipid raft Fyn. If the reporter proteins that carry complementary halves of the luciferase interact, the enzyme can refold in its functional conformation and, upon addition of its substrate, produce luminescence proportionally to the amount of reporter proteins interacting at a given time. N2a cells were seeded in 96-well plates with opaque walls and transparent bottom (PerkinElmer, ViewPlate 96). Cells were transfected with Lipofectamine 2000 according to the manufacturer’s instructions 24 h post-plating with the PCA pair plasmids: phGLuc(1C)-TrkB(WT) or the mutant phGLuc(1C)-TrkB(Y433F) and phGLuc(2 C)-TrkB(WT) for the dimerization experiments; phGLuc(1C)-lipid raft Fyn and phGLuc(2C)-TrkB(WT) or phGLuc(2C)-TrkB(Y433F) for the lipid raft localization experiments. Forty-eight hours post transfection, cells were treated (40 min, 37 °C) in phenol red-free Dulbecco’s modified Eagle medium without serum. For the time course effect of LSD on TrkB dimerization, the medium was changed to phenol red-free Dulbecco’s modified Eagle medium 8 h before the measurement for all conditions, and treatments were added separately at designated timepoints (8 h, 4 h, 2 h and 1 h). The PCA signal was detected with the substrate for the luciferase, native coelenterazine, which was injected into each well directly in the plate reader at a final concentration of 25 mM. One data point was excluded from Fig. 4b by applying Grubb’s method for outlier detection test, which detected it as a significant outlier. This was a pre-established exclusion criteria and a statistical formality, as not excluding the value did not alter the statistical results of the analysis in any significant manner.

### Quantitation of BDNF, TrkB and 5-HT receptor expression by RT–qPCR

N2a cells were transfected to express phGLuc-TrkB using the same protocol as the one described for PCA. Forty-eight hours after transfection, mRNA was extracted using QIAzol Lysis Reagent (Qiagen, 79306). Potential double-strand DNA contamination was removed by dsDNase treatment, and complementary DNA was synthesized using Maxima First Strand cDNA Synthesis Kit (Thermo Fisher Scientific, K1672) following the manufacturer’s protocol. RT–qPCR was carried out in the CFX96 Real-Time PCR System (Bio-Rad) with 0.25 μM of each primer (Extended Data Table 1), using SYBR Green PCR Master Mix (Thermo Fisher Scientific, 4309155). All reactions were run in triplicate for 44 cycles using standard cycling conditions—initial 10 min at 95 °C, followed by 44 cycles of 15 s denaturation at 95 °C, 30 s annealing at 54 °C (for 5-HT_5A_ and 5-HT_1F_ primers) or 65 °C (for all other primers), 30 s extension at 72 °C—and finished with a melting curve analysis (5 s at 65 °C). The sequence of primers^77^ used can be found in Extended Data Table 1. Cortical neurons (DIV21) were treated with DMSO (0.1 %) or LSD (100 nM) for 1 h, 24 h or 72 h. The same protocol was followed as with N2a cells for RNA extraction and cDNA synthesis. For the RT–qPCR, annealing temperature for *Bdnf* was 62 °C, and 63 °C for *Ntrk2* and *Actb. Bdnf* and *Ntrk2* values were normalized to control beta actin (*Actb*). The ΔΔCt method was applied and the fold changes of treatment groups (LSD) over the control groups (vehicle) were analyzed within each timepoint. No template and no reverse transcriptase enzyme controls were included for each reaction. The following primers were used: *Bdnf* total (forward: 5′-GAAGGCTGCAGGGGCATAGACAAA-3′, reverse: 5′-TACACAGGAAGTGTCTATCCTTATG-3′) (ref. ^34^); *Ntrk2* (forward: 5′-GAAGGGAAGTCTGTGACCATTT-3′, reverse: 5′-GTGTGTGGCTTGTTTCATTCAT-3′); *Actb* (forward: 5′-TGTCACCAACTGGGACGATA-3′, reverse: 5′-GGGGTGTTGAAGGTCTCAAA-3′) (ref. ^78^).

### Single-molecule localization microscopy–dSTORM/TIRF

Rat hippocampal neuronal cultures were grown in 1.5H glass coverslip bottom dishes (Ibidi, 81158) and at DIV21 treated with 0.1% DMSO, LSD or PSI (100 nM, 1 h, 37 °C). Then, dishes were fixed with 4% paraformaldehyde (PFA) containing 0.1 µm TetraSpeck beads (Invitrogen, T7279; 1:100). After washing with PBS, dishes were incubated with the blocking buffer (0.1% Triton X-100, 0.05% Tween-20, 10% normal donkey serum, 1% BSA and 0.1% gelatin in PBS) for 1 h at room temperature. Next, dishes were incubated with goat anti-TrkB (R&D Systems, AF1494; 1:100) and rabbit anti-PLCγ1 (Cell Signaling Technologies, 5690S; 1:100) primary antibodies diluted in detergent-free blocking buffer overnight at 4 °C. After washing with PBS, dishes were incubated with Alexa Fluor 647-conjugated donkey anti-goat (Invitrogen, A32849; 1:500) and CF568-conjugated donkey anti-rabbit (Biotium, 20098; 1:500) secondary antibodies in PBS. Dishes were washed and stored in PBS until imaging. For dSTORM/TIRF, we used fresh blinking buffer (0.5 mg ml^−1^ glucose oxidase, 40 µg ml^−1^ catalase, 10% glucose, 10 mM NaCl, 10 mM cysteamine and 50 mM Tris–HCl, pH 8.0). We used a Nikon Eclipse Ti-E N-STORM/TIRF microscope equipped with an ApoTIRF 100×/1.49 numerical aperture (NA) objective, a Perfect Focus System for *Z* focus stabilization, an Andor iXon + 897 back-illuminated EMCCD camera (512 × 512), laser lines 405 nm (100 mW), 561 nm (150 mW) and 647 nm (300 mW) and a 405/488/561/647 nm laser quad band filter cube. Image acquisition was performed with the central 256 × 256 pixels and a total of 20,000 frames (exposure time of 15 ms) for both channels using the NIS-Elements Advanced Research software (Nikon). The 405 nm laser was progressively raised to maintain a constant number of localizations per frame. After chromatic aberration correction by correlation with the fluorescent bead fiducials, fitting of the blinking events to build the single-molecule localization coordinate map and drift correction were performed using the Huygens Localizer 21.04 software (Scientific Volume Imaging). Before identifying particles, the first 512 frames of each channel were discarded, a temporal filter length (factor 30) was applied to determine background signal, and data were prefiltered through the standard deviation of a Gaussian filter (*σ* = 1.0). Next, a threshold calculated from the standard deviation of unfiltered data was multiplied by a factor of 0.75 for the red and 1.5 for the far-red channels and applied to distinguish particles from the background. Pixels from filtered data with values larger than the threshold were considered as candidates for single-molecule localizations. Pixels in filtered data with higher values than surrounding pixels were detected as local maxima. The least-squares fitting was applied for subpixel localization of detected particles, with a fixed full width at half maximum of the Gaussian point spread function of 256 nm. Finally, the resulting single-molecule localization coordinate maps were analyzed with Coloc-Tesseler 1.0 (ref. ^79^) by an experimenter blinded to treatment to compute the Manders colocalization coefficients of TrkB (M^TrkB^) and PLCγ1 (M^PLCγ1^) in dendritic spines on the basis of their overlapping Voronoi diagrams.

### Immunoassays

The phosphorylation of TrkB (pY816) and interaction with PLCγ1 were assessed by enzyme-linked immunosorbent assay (ELISA) as previously described^11,80,81^ with minor modifications. Briefly, white 96-well plates (PerkinElmer, OptiPlate 96F-HB) were precoated with goat anti-TrkB (R&D systems, AF1494; 1:500, overnight, 4 °C) antibody in carbonate buffer (pH 9.8) and blocked with 3% BSA in PBS with 0.1% Tween-20 (PBS-T) for 2 h at room temperature. Next, lysates from rat cortical or hippocampal neuronal cultures (DIV21) that had been treated with DMSO or LSD (1 nM to 100 nM, 1 h, 37 °C) were loaded into the plates. Alternatively, we used lysates containing equal amounts of total protein from PFC or hippocampus dissected from adult mice (WT and Y433F^+/−^) that were killed 1 h after a single intraperitoneal administration of LSD (0.1 mg kg^−1^). Samples were incubated overnight at 4 °C, and 24 h later plates were washed with PBS-T and incubated with either rabbit anti-phospho-TrkB(Tyr816) (Cell Signaling Technology, 4168S; 1:2,000), rabbit anti-PLCγ1 (Cell Signaling Technologies, 5690S; 1:2,000) or rabbit anti-Trk (Cell Signaling Technologies, 92991; 1:2,000) antibodies overnight at 4 °C. After washing with PBS-T, plates were incubated with HRP-conjugated goat anti-rabbit (Bio-Rad, 170–5046; 1:5,000, 1 h, room temperature) antibody, followed by more washing with PBS-T. Finally, the chemiluminescent signal was read after brief incubation with Pierce ECL substrate (Thermo Fisher Scientific, 32106). Luminescence from blank wells that contained all reagents but the sample lysates was used to determine background signal.

The cell surface retention of TrkB was measured as previously described^11,82^. Rat cortical neurons (DIV21) cultivated in clear bottom 96-well plates (PerkinElmer, ViewPlate 96) were treated with DMSO (0.1%) or LSD (100 nM) at different timepoints (5 min, 15 min, 30 min and 1 h) and fixed with 4% PFA. For antagonist experiments, cultures were pretreated with M100907 (100 nM) for 10 min and remained present for the rest of the experiment. After washing with PBS and incubating for 1 h with blocking buffer (5% nonfat dry milk and 5% BSA) at room temperature, plates were incubated with with goat anti-TrkB antibody (R&D Systems, AF1494; 1:1,000, overnight, 4 °C). After further PBS washes, plates were incubated with HRP-conjugated goat anti-rabbit antibody (Bio-Rad, 170–5046; 1:5,000, 1 h, room temperature). Finally, wells were washed with PBS and the chemiluminescent signal was read after brief incubation with Pierce ECL substrate (Thermo Fisher Scientific, 32106).

To determine BDNF protein concentrations, ELISA was run as previously described^83^ with minor modifications. Rat cortical neurons (DIV21) were treated with DMSO (0.1%) or LSD (100 nM) for 5 min, 1 h, 24 h or 72 h, lysed and centrifuged. Samples were diluted in Hanks buffer (125 mM NaCl, 5 mM KCl, 1.2 mM NaH_2_PO_4_, 1 mM CaCl_2_, 1.2 mM MgCl_2_, 1 μM ZnCl_2_, 10 mM glucose, 25 mM HEPES and 0.25% BSA, pH 7.4). Samples and BDNF standards (10–1,000 pg ml^−1^, Peprotech) were acidified at room temperature to pH 3 by adding 1 M HCl and neutralized to pH 7 after 15 min with 1 M NaOH. A Maxisorb Nunc ELISA plate (Thermo Scientific) previously coated with a primary BDNF antibody (1:1,000 in carbonate buffer, overnight, 4 °C) and blocked (2% BSA and 0.1% Triton X-100 in Hanks buffer, 2 h, room temperature). Then, 170 µl of samples and standards were added to the plate with 30 µl of the secondary POD-conjugated BDNF antibody (1:1,000 in 6.66% BSA and 0.66% Triton X-100 in Hanks buffer). The BDNF antibodies were received from Roche and have been previously described^84^. After incubating overnight at 4 °C the plate was washed with PBS-T. BM Blue POD substrate (Roche, 54827-17-1) was added to each well, and the colorimetric reaction was stopped after 20 min by adding 50 µl of 1 M H_2_SO_4_. Absorbance was measured at 490 nm with a plate reader (Varioskan Flash, Thermo Scientific).

### Western blotting

To quantify TrkB protein expression in N2a cells, plates were transfected to express phGLuc-TrkB using the same protocol as described for PCA. Forty-eight hours after transfection, cells were treated with DMSO (0.1 %), LSD (100 nM) or BDNF (10 ng ml^−1^) for 40 min at 37 °C, lysed and centrifuged. To examine the effects of LSD treatment on the phosphorylation of mTOR and ERK, rat cortical neurons (DIV21) were treated with DMSO (0.1%) or LSD (100 nM) for 5 min, 1 h or 24 h. In a separate set of experiments to examine the effect of 5-HT_2A_ activation, M100907 (100 nM) was administered as a 10 min pretreatment and remained in the medium for the rest of the experiment. Next, cells were lysed and centrifuged. In an additional experiment to study the effect of LSD on TrkB interaction with PLCγ1, we used lysates from PFC dissected from adult WT mice that were killed 1 h after a single intraperitoneal administration of LSD (0.1 mg kg^−1^). The co-immunoprecipitation was carried out as previously described^11,23,24^ with minor modifications. Briefly, Magnetic Dynabeads Protein G (Invitrogen, 10004D) were coated with anti-TrkB antibody (R&D Systems, AF1494) at 200 ng of antibody per 1 µl of beads in rotation (2 h, 4 °C). Finally, lysates were mixed with the coated beads (100 µg of total protein per 15 µl of beads) and incubated in rotation (overnight, 4 °C).

Proteins were separated according to their molecular weight by SDS–PAGE and transferred to a polyvinylidene fluoride membrane. Membranes were incubated with the following primary antibodies (all 1:1,000 in 3% BSA in TBS-T, overnight, 4 °C): anti-TrkB (R&D Systems, AF1494), anti-β-actin (Sigma-Aldrich, A1978), anti-phospho-mTOR (Cell Signaling Technology, 5536S), anti-phospho-p44/42 MAPK (Erk1/2) (Cell Signaling Technology, 9106S), anti-mTOR (Cell Signaling Technology, 2972S) and anti-p44/42 MAPK (Erk1/2) (Cell Signaling Technology, 9102S). Membranes were washed with TBS-T and incubated with corresponding secondary HRP-conjugated antibodies (all 1:10,000 in 3% BSA in TBS-T, 1 h, room temperature): HRP-conjugated RaG (Invitrogen, 61-1620), HRP-conjugated GaR (Bio-Rad, 1705046) and HRP-conjugated GaM (Bio-Rad, 170-6515). Protein bands were visualized using enhanced chemiluminescent substrate (Thermo Scientific, 32132, ECL Plus) and imaged with a charge-coupled device camera (G:BOX, Syngene).

### FRAP

FRAP was performed as previously described^11^ with minor modifications. Briefly, hippocampal neurons (DIV16) derived from rat embryos (E18) were grown in glass coverslips and transfected to express GFP-tagged TrkB (WT, Y433F). For FRAP we used a Zeiss LSM 710 confocal microscope equipped with a W PlanApochromat 63×/0.9 NA objective and adapted with an environmental control chamber for live cell imaging set at 37 °C and 5% CO_2_. Twenty-four hours after transfection, coverslips were transferred into pre-acclimatized 35 mm plates containing phenol red-free Hank’s balanced salt solution. Plates were then administered 0.1% DMSO or psychedelics (LSD and PSI) at a final concentration of 100 nM. FRAP was performed during the first hour after treatment. Dendritic spines were localized, and a prebleaching intensity baseline was established. Next, spines were bleached with high laser power until the fluorescence signal decreased to 50% of baseline intensity. Fluorescence recovery in spines was monitored for 1 min after bleaching at a rate of 1 frame per second (f.p.s.). Zen blue 3.2 was used for image analysis. The recovery curve was obtained after subtracting the background and normalizing the bleached spine signal with that of a neighboring area that was not bleached and had equivalent fluorescence intensity at baseline. Finally, the normalized fluorescence intensity values of bleached spines were plotted as percentage of baseline signal and fitted to the exponential one-phase association equation in GraphPad Prism 9.2 to build the recovery curve.

### Spinogenesis

Spinogenesis experiments were performed similarly to previous studies^12,13^ with modifications. Neuronal cultures of WT and Y433F^+/−^ E18 mouse embryos grown in 1.5H glass coverslips were treated with DMSO, LSD or PSI (100 nM) at DIV20. In a separate set of experiments to study the effects of extracellular BDNF and 5-HT_2A_ activation, cultures were also pretreated for 10 min with TrkB.FC (200 ng ml^−1^) or M100907 (100 nM), respectively. TrkB.FC and M100907 remained in the medium for the rest of the experiment. Twenty-four hours later, coverslips were fixed with 4% PFA, washed with PBS, incubated with blocking buffer (0.1% Triton X-100, 0.05% Tween-20, 10% normal donkey serum, 1% BSA and 0.1% gelatin in PBS) for 1 h at room temperature and then overnight with rabbit anti-MAP2 (Invitrogen, PA5-17646; 1:100) primary antibody at 4 °C. After washing with PBS, coverslips were incubated with CF568-conjugated donkey anti-rabbit (Biotium, 20098; 1:500) for 1 h at room temperature. Another round of PBS washes was followed by incubation with Alexa Fluor 647-conjugated phalloidin (Invitrogen, A22287; 1:40) overnight at 4 °C. Next, coverslips were briefly washed in phosphate buffer (PB) and mounted on slides with ProLong Glass Antifade Mountant (Invitrogen, P36982). We used a Zeiss LSM 880 confocal microscope with an AiryScan GaAsP detector equipped with a PlanApochromat 63×/1.4 NA objective. We used the BP 495–550 + LP 570 AiryScan filter and Zen Black in super-resolution mode for image acquisition. Pinhole size was adapted accordingly, and frame size was set to optimal. Sensors were set to continuous adjustment across the imaging sessions to ensure optimal sensor alignment and signal detection. Raw data were processed into AiryScan images with the autofilter mode on using Zen Blue. Finally, image analysis was performed in ImageJ by experimenters blinded for treatment and genotype. Spines were quantified in 15-µm-long dendrite sections.

### Dendritogenesis

Neurons from WT or Y433F^+/−^ mouse embryo (E18) were plated and treated with 100 nM of psychedelics (LSD and PSI) or vehicle (0.1% DMSO) once a day for three consecutive days starting at DIV5 (cumulative doses). In a separate set of experiments to study the effects of extracellular BDNF and 5-HT_2A_ activation, cultures were also pretreated for 10 min with TrkB.FC (200 ng ml^−1^) or M100907 (100 nM), respectively. Similar to treatment with the psychedelics, TrkB.FC and M100907 pretreatments were administered in cumulative doses and remained in the medium for the rest of the experiment. At DIV7, neurons were pre-incubated for 1 h with 10 μM MgCl_2_ to prevent excitotoxicity, then neurons were transfected for the heterologous expression of mCherry. To minimize any possible interference of the transfection protocol upon the drug effect, transfection was done in the morning while treatment with psychedelics was always added in the evening. Twenty-four hours after transfection, neurons were fixed with 4% PFA for 20 min at room temperature, washed in PBS-T, blocked for 30 min with 3% BSA in PBS-T and incubated with chicken anti-MAP2 (Abcam, ab5392; 1:5,000, 1 h, room temperature). Then, coverslips were washed in PBS-T and incubated with Alexa Fluor 647-conjugated antibody goat anti-chicken (Invitrogen, A-21449; 1:1,000, 1 h, room temperature). Next, coverslips were washed in PBS-T and MilliQ water, and mounted in Dako fluorescence mounting medium (Agilent, S302380-2). Ten optical sections with a *Z*-step of 1.3 μm were acquired using a Zeiss LSM 710 confocal microscope with a LCI Plan/Neofluar 25×/0.8 lmm Korr DIC M27 objective. In the mCherry channel, the center of the soma was manually assigned and the number of dendritic intersections at fixed distances (every 5 μm) from the soma was automatically counted using the Sholl analysis plugin of ImageJ.

### Long-term neuronal survival and BrdU immunostaining

WT and Y433F^+/−^ male mice were administered four doses of 5-bromo-2′-deoxyuridine (BrdU, 75 mg kg^−1^ per injection, every 2 h) intraperitoneally to tag newborn neurons in the hippocampus and returned to their home cage. Twenty-four hours later, animals were injected a single dose of saline or LSD (0.1 mg kg^−1^) intraperitoneally. Four weeks later, animals were intracardially perfused with PBS followed by 4% PFA. Brains were dissected and postfixed with 4% PFA (overnight, 4 °C). Next, brains were transferred to 30% sucrose (0.1% PBS) solution and stored at 4 °C for 48–72 h. Brains were moved into disposable vinyl specimen molds (Sakura, Tissue-Tek Cryomold standard) and covered in Tissue-Tek O.C.T. Compound (Sakura). Trays were placed on dry ice and brains covered once more in TissueTec to ensure full coverage. Once frozen, samples were stored at −80 °C. Brains were sliced coronally at 40 µm thickness, collecting every sixth slice. Slices were stored in cryoprotectant fluid (30% sucrose, 30% ethylene glycol, 1% PVP-40, and 0.05 M PB) in −20 °C until staining. Every third hippocampal slice containing dentate gyrus (DG) was collected for washing with 0.1 M PBS and staining. BrdU immunofluorescence was conducted similarly as previously reported in the literature^28^ with modifications. Hippocampal slices were incubated in 2.0 M HCl (30 min, 37 °C) and washed with PBS. Slices were then incubated in 0.1 M borate buffer (pH 8.0) at room temperature for 10 min, followed by further washes in PBS and 1 h incubation in 0.1% PBS blocking buffer (0.5% Triton X-100, 5% normal donkey serum, 5% normal goat serum, 1% BSA and 0.1% gelatin). Sections were incubated with primary antibodies rat anti-BrdU (Abcam, ab6326; 1:200) and mouse anti-NeuN (Millipore, MAB377; 1:1,000) for 48 h at 4 °C with gentle shaking. Slices were then washed with PBS, followed by incubation with secondary antibodies Alexa Fluor 488 donkey anti-rat (Invitrogen, A21208; 1:300) and Alexa Fluor 568 goat-anti-mouse (Invitrogen, A11004; 1:500) for 2 h at room temperature and protected from light. Slices were washed in PBS and PB before mounting with Dako fluorescence mounting medium (Agilent, S302380-2) and slides stored at 4 °C until imaging. A total of eight slices containing both hemispheres and covering the whole DG (every third slice) were imaged with a Zeiss LSM 710 confocal microscope. Fifteen optical sections with a *Z*-step of 2 µm were acquired per slice with a PlanApochromat 10×/0.45 M27. BrdU^+^ and BrdU^+^/NeuN^+^ cells in the DG were counted manually by experimenters blinded to treatment and genotype using ZEN Blue 3.2. Similarly to Ma et al.^28^, we estimated the number of mature neurons (BrdU^+^/NeuN^+^) in the DG of both hemispheres by multiplying by 6 the total count derived from every third hippocampal slice. The percentage of mature neurons over total BrdU^+^ cells in the DG was also calculated.

### OD plasticity

Female and male mice were 17 weeks old at the time of monocular deprivation, since the physiological critical period in the binocular region of the visual cortex is closed by then^85^. Transparent skull surgeries were executed as described before^11,86,87^. Briefly, mice were injected with an anesthetic mixture containing 0.05 mg kg^−1^ fentanyl (Hameln), 5 mg kg^−1^ midazolam (Hameln) and 0.5 mg kg^−1^ medetomidine (Orion Pharma) diluted in saline. Eyes were protected from drying with eye-protective gel (Alcon), and the head was shaved and fixed in a stereotaxic frame with a heating pad maintained at 37 °C. A mixture of lidocaine and adrenaline (Orion Pharma) was applied locally on the head, and the scalp above the visual cortex was cut. The periosteum was removed gently, and the skull was rapidly cleaned from fat with acetone. The surface of the skull was covered with a thin layer of cyanoacrylate glue (Henkel, Loctite 401), followed by two layers of acryl, obtained by mixing colorless acrylic powder (EUBECOS) with liquid methacrylate (Densply). Mice were awakened with 1 mg kg^−1^ atipamezole (VetMedic) and postoperative analgesia provided with 0.1 mg kg^−1^ buprenorphine (Indivior) and 5 mg kg^−1^ carprofen (VetScan). After 24 h, mice were anesthetized with isoflurane and the acryl polished. A metal bar holder was glued and fixed with a mixture of cyanoacrylate glue and dental cement (Densply) to the skull, and transparent nail polish (Electron Microscopy Sciences, 72180) was applied above the visual cortex, which was visible at the center of the holder. Monocular deprivation lasted for 1 week. Mice were anesthetized with isoflurane, antibiotic gel (Dechra, Isathal Vet 1%) was applied to the eye contralateral to the hemisphere being imaged, eyelashes were cut and eye was sutured shut with three mattress sutures (Ethiconn). Antibiotic ointment (Anten, Oftan Dexa-Chlora) was applied on the sutured eye and 5 mg kg^−1^ carprofen was injected subcutaneously for postoperative analgesia. All animals were checked daily, and the sutured eye was opened right before the second imaging. Mice showing corneal injury were excluded from experiments. LSD (0.1 mg kg^−1^) was administered intraperitoneally three times on alternate days during the week-long monocular deprivation period following previously described protocols^11,86,87^. Optical imaging of the intrinsic signal was performed to assess visually evoked cortical activity by measuring the changes in the oxygen saturation of hemoglobin in the primary visual cortex^86,88,89^. Briefly, two imaging sessions were performed: one before LSD administration and monocular deprivation (IOS I) and a second session at the end of monocular deprivation (IOS II). Anesthesia was induced with 1.8% isoflurane and maintained with 1.2% isoflurane in a 1:2 mixture of O_2_:air. Animals were kept on a heating pad, 25 cm in front of the stimulus monitor, where a 2° horizontal bar moving upwards was shown with temporal and spatial frequency of 0.125 Hz and 1/80 degree, respectively. The visual stimulus was limited to the central part of the monitor (width: −15° to 5° azimuth, relative to the animal visual field) to stimulate only the binocular part of the visual field. The continuous–periodic stimulation was synchronized with a continuous frame acquisition, collected independently for each eye^88^. To acquire a vascular map, the skull was illuminated with a green light (540 ± 20 nm). The camera was then focused 600 µm below the cortical surface, and a red light (625 ± 10 nm) was used to record the intrinsic signal. The signal was recorded from one eye at a time, with the other eye covered by a patch. The continuous–periodic stimulation was synchronized with a continuous frame acquisition, and frames were collected at a rate of 30 f.p.s. for 5 min and stored as a 512 × 512 pixel image. Following the acquisition for both contralateral (C) and ipsilateral (I) eyes, the cortical maps were computed after spatial binning and thresholding by using a custom-made analysis software package that performs Fourier decomposition on the cortical maps^88^. The OD score as (C − I)/(C + I) was calculated, and the ocular dominance index (ODI) was determined as the mean of the OD score for all responsive pixels, as described by Cang et al.^89^. The ODI values range between −1 and +1, where positive values correspond to a contralateral bias and negative ones to ipsilateral bias. ODI values of 0 indicate that inputs from ipsilateral and contralateral eyes are equally strong.

### Head-twitch response

WT and Y433F^+/−^ mice were administered a single intraperitoneal dose of saline or LSD (0.1 mg kg^−1^). Another independent cohort of WT mice received a single intraperitoneal injection of M100907 (0.5 mg kg^−1^) or vehicle followed 30 min later by another injection of LSD (0.1 mg kg^−1^) or vehicle^90^. Immediately after the last injection, the animals were placed inside a transparent chamber and their activity video-recorded for 30 min. Similarly to previous studies^91^, head twitches were scored as counts every 2 min by observers blinded to treatments and genotypes.

### rFST

rFST was conducted in WT and Y433F^+/−^ mice as described in literature^33,92^ with minor modifications. Briefly, female and male adult mice were exposed to five consecutive pretest sessions (5 min of duration, 24 h apart). In the first experiment, WT or Y433F^+/−^ mice received a single intraperitoneal injection of LSD (0.1 mg kg^−1^) or vehicle 24 h after the last pretest session. In a second independent experiment, WT mice received an intraperitoneal injection of M100907 (0.5 mg kg^−1^) or vehicle 24 h after the last pretest session, followed by an intraperitoneal injection of LSD (0.1 mg kg^−1^) or saline 30 min later. The animals were submitted to a 5 min test session 7 days after the drug administration. The apparatus consisted of 5 l glass beaker cylinders (19 cm diameter, 20 cm water column at 23 ± 2 °C). The water was changed between each trial, for each animal. After each session, the animals were dried and kept in a warmed cage before returning to their home cages. The pretest and test sessions were recorded and analyzed by a trained observer blind to treatment and genotype.

### Contextual fear conditioning and fear reinstatement

Male WT mice (C57BL/6JRccHsd, Envigo) were used for contextual fear conditioning experiments with and without extinction training as previously described^11,34^ with modifications. Briefly, animals were conditioned in context A (metallic bar flooring, transparent Plexiglass walls, 23 × 23 × 35 cm arena) with a series of five scrambled foot shocks (0.6 mA, 2 s) along a 480 s session. Twenty-four hours later, animals were administered a single dose of saline or LSD (0.1 mg kg^−1^) intraperitoneally and returned to their home cage. During the following 3 days, mice were exposed to extinction training in context A for 480 s every 24 h or placed for the same duration and number of sessions in context B (black planar floor, black nontransparent walls, 23 × 23 × 35 cm arena), which we categorized as extinction and no extinction trainings, respectively. Afterwards, animals were placed back into their home cage and left undisturbed for 3 weeks. Finally, all mice were reconditioned with a new set of five scrambled foot shocks (0.6 mA, 2 s) along a 480 s session in context A, and their fear reinstatement response was measured 24 h later for the same duration also in context A. All sessions were conducted under constant illumination (100 lux). For the contextual fear conditioning and fear reinstatement experiment with male WT and Y433F^+/−^, we used the same protocol as for animals undergoing extinction training in context A above. Baseline freezing was established during the first conditioning session. Movement was measured by infrared beams placed on the sides of the conditioning chamber, and freezing was automatically determined by the fear conditioning apparatus and software (TSE Systems) and expressed as the percentage of time spent freezing.

### Statistics

Data were analyzed with GraphPad Prism 9.2. Detailed statistical information for all presented data is available in Supplementary Table 1. No statistical methods were used to predetermine sample sizes, but our sample sizes are similar to those reported in previous publications^11^. Data met the assumptions for normality and equal variances for the statistical tests used, and when this was not the case, data were assumed to be normal and individual data points are displayed. Sample and animal distribution among groups was randomized. Single comparisons among groups were made using Student’s *t*-test (two-tailed, paired or unpaired), and one-way or two-way analysis of variance followed by Bonferroni’s post hoc test was used for multiple comparisons unless indicated otherwise. Data are presented as mean ± standard error of the mean (s.e.m.) and *P < 0.05, unless otherwise indicated.

### Reporting summary

Further information on research design is available in the Nature Portfolio Reporting Summary linked to this article.

## Online content

Any methods, additional references, Nature Portfolio reporting summaries, source data, extended data, supplementary information, acknowledgements, peer review information; details of author contributions and competing interests; and statements of data and code availability are available at 10.1038/s41593-023-01316-5.

### Supplementary information

Supplementary Information Supplementary Table 3. Simulated system conditions.

Reporting Summary

Supplementary Table 1 Supplementary Table 1. Detailed statistical summary.

Supplementary Table 2 Supplementary Table 2. MS of HEK293T lysates.

## Figures and Tables

**Extended Data Table 1:**
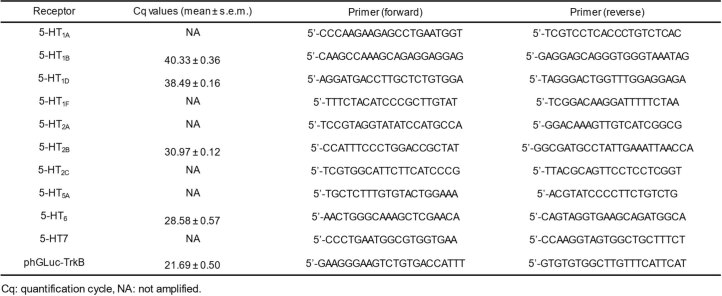
N2a cells do not express 5-HT_2A_ receptors

Table showing mRNA expression of 5-HT receptors and lack of 5-HT_2A_ in N2a cells. Data shown as mean ± s.e.m.

## Data Availability

All experimental data used in the present study are available at FigShare (https://figshare.com/s/cfa23c9c3a7b4f16610d). Y433F^+/−^ mice are available upon request from the authors.

## Code availability

This study did not generate new code.
