# Supplementary material for: Psychedelics promote plasticity by directly binding to BDNF receptor TrkB
Source: Nat Neurosci. 2023 Jun 5;26(6):1032–41. doi: 10.1038/s41593-023-01316-5 (PMC10244169; doi:10.1038/s41593-023-01316-5)
Supplement: Supplementary file 1 — Supplementary Table 3. Simulated system conditions. [file 41593_2023_1316_MOESM1_ESM.pdf]

nature neuroscience

Article https://doi.org/10.1038/s41593-023-01316-5

Psychedelics promote plasticity by directly binding to BDNF receptor TrkB

In the format provided by the authors and unedited

Supplementary Table 3. Simulated systems. The table lists the variants of TM TrkB dimers embedded in POPC-CHOL lipid membranes, mole percentage of cholesterol (ρ_{CHOL}), the number of POPC (N_{POPC}) and cholesterol (N_{CHOL}) molecules in a membrane, the number of independent simulation repeats (N_{sim}), and the simulation length per repeat (t_{sim}). "WT", "Y433F.homo", and "V437A.homo" refer in respective order to the wild-type and homozygous Y433F and V437A variants of a TrkB TM dimer (residues 427-459). LSD, LSD^+: psychoactive (+)-LSD (in a 5R,8R configuration) in a neutral and protonated form. PSI, PSI^+: psilocin in a neutral and protonated form. Lisuride, Lisuride+: lisuride in a neutral and protonated form. CAR: cabergoline in a neutral form (pKa = 6.22). DHE: dihydroergotamine in a neutral form (pKa = 6.75).

System name	Protein variant	Drug type	ρ_{CHOL} (mol%)	N_{POPC}	N_{CHOL}	N_{sim}	t_{sim} (μs)
System 1[#]	WT	LSD	20	112	28	100	1
System 2[∇]	WT	LSD	20	112	28	20	2
System 3[∇]	WT	LSD^+	20	112	28	20	2
System 4[∇]	WT	PSI	20	112	28	20	2
System 5[∇]	WT	PSI^+	20	112	28	20	2
System 6[∇]	WT	Lisuride	20	112	28	20	2
System 7[∇]	WT	$Lisuride^+$	20	112	28	20	2
System 8[∇]	WT	CAR	20	112	28	20	2
System 9[∇]	WT	DHE	20	112	28	20	2
System 10[∇]	Y433F	LSD	20	112	28	20	2
System 11[∇]	V437A	LSD	20	112	28	20	2
System 12[∇]	Y433F	LSD^+	20	112	28	20	2
System 13[∇]	V437A	LSD^+	20	112	28	20	2
System 14[∇]	Y433F	PSI^+	20	112	28	20	2
System 15[∇]	V437A	PSI^+	20	112	28	20	2
System 16[†]	WT	LSD	20	112	28	1	0.06
System 17[†]	Y433F	LSD	20	112	28	1	0.06
System 18[†]	V437A	LSD	20	112	28	1	0.06
System 19[†]	WT	LSD^+	20	112	28	1	0.06
System 20[†]	Y433F	LSD^+	20	112	28	1	0.06
System 21[†]	V437A	LSD^+	20	112	28	1	0.06
System 22[†]	WT	PSI	20	112	28	1	0.06
System 23[†]	WT	PSI^+	20	112	28	1	0.06
System 24[†]	Y433F	PSI^+	20	112	28	1	0.06
System 25[†]	V437A	PSI^+	20	112	28	1	0.06
System 26[†]	WT	Lisuride	20	112	28	1	0.06
System 27[†]	WT	$Lisuride^+$	20	112	28	1	0.06
System 28[†]	WT	CAR	20	112	28	1	0.16
System 29[†]	WT	DHE	20	112	28	1	0.06
System 30[§]	WT	-	40	90	60	10	1
System 31[§]	WT	LSD	40	90	60	10	1
System 32[§]	WT	LSD^+	40	90	60	10	1
System 33[§]	WT	PSI^+	40	90	60	10	1
System 34[§]	WT	$Lisuride^+$	40	90	60	10	1

Simulations to explore LSD binding site and mode.

∇ Simulations to optimize the binding modes of each drug in the wild type and mutated TM TrkB dimers.

† FEP/HREX (free energy) simulations to estimate the drug binding affinities.

§ Simulations to explore the effect of cholesterol and the drugs on the conformation of the protein.
