# Supplementary material for: Psychedelics promote plasticity by directly binding to BDNF receptor TrkB
Source: Nat Neurosci. 2023 Jun 5;26(6):1032–41. doi: 10.1038/s41593-023-01316-5 (PMC10244169; doi:10.1038/s41593-023-01316-5)
Supplement: Supplementary file 2 — Reporting Summary [file 41593_2023_1316_MOESM2_ESM.pdf]

nature portfolio

Corresponding author(s): Eero Castrén, Plinio Casarotto and Ilpo Vattulainen

Last updated by author(s): Feb 17, 2023

Reporting Summary

Nature Portfolio wishes to improve the reproducibility of the work that we publish. This form provides structure for consistency and transparency in reporting. For further information on Nature Portfolio policies, see our Editorial Policies and the Editorial Policy Checklist.

Statistics

For all statistical analyses, confirm that the following items are present in the figure legend, table legend, main text, or Methods section.

n/a	Confirmed	
☐	☒	The exact sample size (*n*) for each experimental group/condition, given as a discrete number and unit of measurement
☒	☐	A statement on whether measurements were taken from distinct samples or whether the same sample was measured repeatedly
☐	☒	The statistical test(s) used AND whether they are one- or two-sided<br>*Only common tests should be described solely by name; describe more complex techniques in the Methods section.*
☒	☐	A description of all covariates tested
☐	☒	A description of any assumptions or corrections, such as tests of normality and adjustment for multiple comparisons
☐	☒	A full description of the statistical parameters including central tendency (e.g. means) or other basic estimates (e.g. regression coefficient) AND variation (e.g. standard deviation) or associated estimates of uncertainty (e.g. confidence intervals)
☐	☒	For null hypothesis testing, the test statistic (e.g. *F*, *t*, *r*) with confidence intervals, effect sizes, degrees of freedom and *P* value noted<br>*Give P values as exact values whenever suitable.*
☒	☐	For Bayesian analysis, information on the choice of priors and Markov chain Monte Carlo settings
☒	☐	For hierarchical and complex designs, identification of the appropriate level for tests and full reporting of outcomes
☒	☐	Estimates of effect sizes (e.g. Cohen's *d*, Pearson's *r*), indicating how they were calculated

Our web collection on statistics for biologists contains articles on many of the points above.

Software and code

Policy information about availability of computer code

Data collection	Data and code availability statements have been included, and software used for data collection is described in detail. dSTORM/TIRF image acquisition with NIS-Elements Advanced Research 4.50 with 6D image acquisition module and JOBS high content module.
Data analysis	Data analysis was performed using GraphPad Prism 9.2.<br>Image analysis was performed using FIJI 1.51, Huygens Localizer 21.04 software (Scientific Volume Imaging) or Coloc-Tesseler 1.0. TALOS-N software used to determine the secondary structure of the peptide for NMR chemical shift data.

For manuscripts utilizing custom algorithms or software that are central to the research but not yet described in published literature, software must be made available to editors and reviewers. We strongly encourage code deposition in a community repository (e.g. GitHub). See the Nature Portfolio guidelines for submitting code & software for further information.

Data

Policy information about availability of data

All manuscripts must include a data availability statement. This statement should provide the following information, where applicable:

- Accession codes, unique identifiers, or web links for publicly available datasets
- A description of any restrictions on data availability
- For clinical datasets or third party data, please ensure that the statement adheres to our policy

All experimental data used in the present study are available in FigShare (https://figshare.com/s/cfa23c9c3a7b4f16610d) as stated in the Data Availability section.

Field-specific reporting

Please select the one below that is the best fit for your research. If you are not sure, read the appropriate sections before making your selection.

☒ Life sciences ☐ Behavioural & social sciences ☐ Ecological, evolutionary & environmental sciences

For a reference copy of the document with all sections, see nature.com/documents/nr-reporting-summary-flat.pdf

Life sciences study design

All studies must disclose on these points even when the disclosure is negative.

Sample size	Sample sizes for in vitro and in vivo experiments were estimated based on similar experiments previously described in literature (Casarotto, P. C. et al. Antidepressant drugs act by directly binding to TRKB neurotrophin receptors. Cell 184, 1299–1313.e19 (2021)).
Data exclusions	One data point was excluded from fig 4b by applying Grubb's method for outlier detection test (https://www.graphpad.com/support/faqid/1598/), which detected it as a significant outlier. This was a pre-established exclusion criteria and a statistical formality, as not excluding the value did not alter the statistical results of the analysis in any significant way (only one outlier data point was excluded from a total of n per group = 24).
Replication	All experiments were conducted in at least 3 separate and independent cohorts. No difference was observed between the cohorts, therefore we assume all replications were aligned.
Randomization	For in vitro approaches, treatment was randomized over the plates and analyzed blindly.<br>For in vivo approaches, all methods were automated and conducted by an experimenter blind to the treatments. Animals were also randomly assigned to treatment groups before the start of the experiments.
Blinding	All experiments were conducted by two experimenters, one of the investigators was responsible for the treatments while the other was solely responsible for the analysis. Only after the final collection of data, the treatment codes were broken.

Reporting for specific materials, systems and methods

We require information from authors about some types of materials, experimental systems and methods used in many studies. Here, indicate whether each material, system or method listed is relevant to your study. If you are not sure if a list item applies to your research, read the appropriate section before selecting a response.

Materials & experimental systems

n/a	Involved in the study	
☐	☒	Antibodies
☐	☒	Eukaryotic cell lines
☒	☐	Palaeontology and archaeology
☐	☒	Animals and other organisms
☒	☐	Human research participants
☒	☐	Clinical data
☒	☐	Dual use research of concern

Methods

n/a	Involved in the study	
☒	☐	ChIP-seq
☒	☐	Flow cytometry
☒	☐	MRI-based neuroimaging

Antibodies

Antibodies used

anti-TRKB (R&D systems, AF1494); https://www.rndsystems.com/products/human-mouse-rat-trkb-antibody_af1494
anti-PLCγ1 (Cell Signaling Technologies, 5690S); https://www.cellsignal.com/products/primary-antibodies/plcg1-d9h10-xp-rabbit-mab/5690
anti-phospho-TrkB(Tyr816) (Cell Signaling Technology, 4168S); https://www.cellsignal.com/products/primary-antibodies/phospho-trka-tyr785-trkb-tyr816-c67c8-rabbit-mab/4168
anti-TRK (Cell Signaling Technologies, 92991); https://www.cellsignal.com/products/primary-antibodies/trk-pan-a7h6r-rabbit-mab/92991
anti-phospho-mTOR (Cell Signaling Technology, 5536S); https://www.cellsignal.com/products/primary-antibodies/phospho-mtor-ser2448-d9c2-xp-rabbit-mab/5536
anti-phospho-p44/42 MAPK (Erk1/2) (Cell Signaling Technology, 9106S); https://www.cellsignal.com/products/primary-antibodies/phospho-p44-42-mapk-erk1-2-thr202-tyr204-e10-mouse-mab/9106
anti-mTOR (Cell Signaling Technology, 2972S); https://www.cellsignal.com/products/primary-antibodies/mtor-antibody/2972
anti-p44/42 MAPK (Erk1/2) (Cell Signaling Technology, 9102S); https://www.cellsignal.com/products/primary-antibodies/p44-42-mapk-erk1-2-antibody/9102
anti-MAP2 (Invitrogen, PA5-17646); https://www.thermofisher.com/antibody/product/MAP2-Antibody-Polyclonal/PA5-17646
anti-MAP2 (Abcam, ab5392); https://www.abcam.com/map2-antibody-ab5392.html
anti-BrdU (Abcam, ab6326); https://www.abcam.com/brdu-antibody-bu175-icr1-proliferation-marker-ab6326.html

anti-NeuN (Millipore, MAB377, clone A60);
anti-β-actin (Sigma-Aldrich, A1978);
Alexa Fluor 488 donkey anti-rat (Invitrogen, A21208);
Alexa Fluor 647-conjugated antibody goat anti-chicken (Invitrogen, A-21449);
Alexa Fluor 647-conjugated donkey anti-goat (Invitrogen, A32849);
Alexa Fluor 568 goat-anti-mouse (Invitrogen, A11004);
CF568-conjugated donkey anti-rabbit (Biotium, 20098);
HRP-conjugated GaR (BioRad, 170-5046);
HRP-conjugated RaG (Invitrogen, 61-1620);
HRP-conjugated GaM (Bio-Rad, 170-6515)

Validation

All these antibodies were validated by the manufacturer and used in previous studies from our laboratory (e.g. Casarotto, P. C. et al. Antidepressant drugs act by directly binding to TRKB neurotrophin receptors. Cell 184, 1299–1313.e19 (2021)). Please see more information on validation and more references for each primary antibody following the manufacturer's link in the section above.

Eukaryotic cell lines

Policy information about cell lines

Cell line source(s)

HEK293T and N2A cell lines were used. Available upon request from the authors and also from commercial sources (e.g. https://www.abcam.com/human-wild-type-hek-293t-cell-line-ab255593.html or https://www.abcam.com/mousewild-type-neuro-2a-cell-line-ab279975.html)

Authentication

These cell lines were not authenticated

Mycoplasma contamination

These cell lines were not assayed for mycoplasma contamination

Commonly misidentified lines
(See ICLAC register)

No commonly misidentified lines were used in this study.

Animals and other organisms

Policy information about studies involving animals; ARRIVE guidelines recommended for reporting animal research

Laboratory animals

The C57BL/6NTac-NTrk2em6006 strain carrying the point mutation in TrkB (Y433F) was maintained in C57BL/6JRccHsd to generate WT and Y433F heterozygous mice. The exact age and sex of all mice used in this study is specified in the corresponding Methods section. We used both female and male mice that were at least 10 weeks old at the start of experiments for this study. Animals were caged in standard housing conditions as described in the Animals section in Methods.

Wild animals

No wild animals were used in this study.

Field-collected samples

No field-collected samples were used in this study.

Ethics oversight

All animal experiments were performed in compliance with institutional guidelines and approved by the Regional State Administrative Agency for Southern Finland (ESAVI/38503/2019).

Note that full information on the approval of the study protocol must also be provided in the manuscript.
